# In Situ Transmission Electron Microscopy of Electrocatalyst Materials: Proposed Workflows, Technical Advances, Challenges, and Lessons Learned

**DOI:** 10.1002/smtd.202400851

**Published:** 2024-12-20

**Authors:** Ahmed M. Abdellah, Kholoud E. Salem, Liza‐Anastasia DiCecco, Fatma Ismail, Amirhossein Rakhsha, Kathryn Grandfield, Drew Higgins

**Affiliations:** ^1^ Department of Chemical Engineering McMaster University Hamilton ON L8S 4L7 Canada; ^2^ Canadian Centre for Electron Microscopy McMaster University Hamilton ON L8S 4M1 Canada; ^3^ Department of Materials Science and Engineering McMaster University Hamilton ON L8S 4L8 Canada; ^4^ Department of Biomedical Engineering The Pennsylvania State University University Park PA 16802 USA; ^5^ School of Biomedical Engineering McMaster University Hamilton ON L8S 4L7 Canada

**Keywords:** electrocatalysis, electrochemical CO_2_ conversion, in situ characterization, in situ liquid‐phase transmission electron microscopy, operando characterization, workflow

## Abstract

In situ electrochemical liquid phase transmission electron microscopy (LP‐TEM) measurements utilize micro‐chip three‐electrode cells with electron transparent silicon nitride windows that confine the liquid electrolyte. By imaging electrocatalysts deposited on micro‐patterned electrodes, LP‐TEM provides insight into morphological, phase structure, and compositional changes within electrocatalyst materials under electrochemical reaction conditions, which have practical implications on activity, selectivity, and durability. Despite LP‐TEM capabilities becoming more accessible, in situ measurements under electrochemical reaction conditions remain non‐trivial, with challenges including electron beam interactions with the electrolyte and electrode, the lack of well‐defined experimental workflows, and difficulty interpreting particle behavior within a liquid. Herein a summary of the current state of LP‐TEM technique capabilities alongside a discussion of the relevant experimental challenges researchers typically face, with a focus on in situ studies of electrochemical CO_2_ conversion catalysts is provided. A methodological approach for in situ LP‐TEM measurements on CO_2_R catalysts prepared by electro‐deposition, sputtering, or drop‐casting is presented and include case studies where challenges and proposed workflows for each are highlighted. By providing a summary of LP‐TEM technique capabilities and guidance for the measurements, the goal is for this paper to reduce barriers for researchers who are interested in utilizing LP‐TEM characterization to answer their scientific questions.

## Graphical abstract

This paper introduces the capabilities of in situ electrochemical liquid‐phase TEM (LP‐TEM) to study electrochemical CO_2_ reduction catalysts. A methodological workflow is presented alongside case studies of relevant catalysts which covers sample preparation, experimental setup, and data analysis. Key challenges are discussed, and strategies are presented to make in situ LP‐TEM accessible for researchers with scientific questions about electrocatalytic materials and processes.

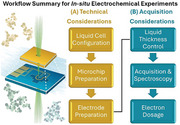

## 1 Introduction

The continuous accumulation of atmospheric carbon dioxide (CO_2_) levels is a primary cause of global warming and associated climate change concerns that have far‐reaching consequences on our ecosystem and society.^[^ ^1^, ^2^, ^3^ ^]^ These escalating issues highlight an urgent need to find effective technologies for transitioning the energy sector away from fossil fuels and toward sustainability.^[^ ^4^, ^5^, ^6^, ^7^ ^]^ Amongst them, electrochemical CO_2_ reduction (CO_2_R) technologies can provide a route to produce valuable chemicals and fuels using low‐carbon electricity as the energy input.^[^ ^4^, ^5^, ^8^, ^9^ ^]^ However, low reaction rates and selectivity owing to the thermodynamic stability and complicated multi‐step CO_2_R mechanisms lead to poor energy conversion efficiencies, which alongside limited catalyst and system stability restrain the widespread deployment of CO_2_R technologies.^[^ ^9^, ^10^ ^]^ New catalyst material developments with improved performance and stability are required, which can be informed by an advanced understanding of CO_2_R catalyst performance descriptors and degradation mechanisms. Of particular use would be scientific insight into the structure, properties, and local reactive environments of the catalyst materials at the nanoscale and how they change as a function of electrode potential and time.^[^ ^5^ ^]^ To this end, in situ and operando spectroscopic characterization methods provide means to investigate catalytic systems, material structures, and reaction processes under realistic experimental conditions.^[^ ^5^, ^8^, ^11^, ^12^ ^]^ Technically speaking, ‘in situ’ characterization refers to measurements performed under relevant reaction conditions, meanwhile ‘operando’ characterization (a sub‐class of in situ characterization) refers to measurements performed on materials under realistic operating conditions where reaction rates and selectivities are simultaneously being measured. It is important to note that the term operando is commonly used incorrectly in the literature, and to this end, in this article, we stick to using in situ as our terminology of choice as it captures both in situ and truly operando measurements. Techniques such as in situ X‐ray absorption spectroscopy (XAS), in situ X‐ray photoelectron spectroscopy (XPS), and in situ infrared spectroscopy (IR) have been used for studying CO_2_R processes, providing scientific knowledge into material properties and near‐surface processes occurring under reaction conditions.^[^ ^13^, ^14^, ^15^, ^16^, ^17^, ^18^, ^19^, ^20^, ^21^, ^22^ ^]^ However, these techniques do not directly characterize the catalyst morphology that is known to change under reaction conditions and provide very little (or no) spatially resolved insight.^[^ ^13^, ^23^, ^24^ ^]^

Alternatively, spectro‐microscopy^[^ ^25^, ^26^ ^]^ and microscopy techniques^[^ ^9^, ^27^, ^28^, ^29^, ^30^, ^31^, ^32^, ^33^ ^]^ offer an in situ/operando route for tracking real‐time morphological changes in catalyst materials. Transmission electron microscopy (TEM) in particular can provide a high spatial resolution under reaction conditions, sometimes approaching the atomic level.^[^ ^13^, ^34^, ^35^ ^]^ However, one critical challenge in probing solid‐liquid interfaces (i.e., catalyst/electrolyte interfaces) in CO_2_R systems is the inherent difficulty in directly imaging atomic‐scale dynamics in the presence of the liquid electrolyte that limits spatial resolution due to electron scattering. To address this challenge, recent studies have focused on minimizing measurement noise, enhancing the resolution, and reducing the thickness of the liquid electrolyte.^[^ ^22^, ^36^, ^37^ ^]^ Moreover, the development of new electrochemical TEM holders that offer more controlled environments could enhance the ability to probe catalyst/electrolyte interfaces in real‐time.^[^ ^13^, ^38^, ^39^, ^40^ ^]^ For in situ measurements under electrochemical conditions, a fundamental limitation will be for the electrode to maintain ionic contact with the electrolyte (i.e., the electrode surface will need to form an interface with the liquid or polymer electrolyte). Achieving this while minimizing or event‐eliminating electron scattering by the electrolyte could potentially be achieved by introducing unique in situ TEM cell designs, or creative approaches to form a catalyst/electrolyte interface that does not adversely impact the resolution of the measurements.^[^ ^40^ ^]^ Furthermore, modern TEMs are equipped with analytical spectroscopic tools, such as energy dispersive X‐ray analysis (EDX), electron diffraction, and electron energy loss spectroscopy (EELS), that can provide a complementary understanding of spatially resolved compositions, phase, and electronic structures. Leveraging recent technological advancements, liquid phase‐TEM (LP‐TEM) has gained popularity owing to its ability to provide real‐time imaging of morphological changes and dynamic processes in nanostructured materials in liquid environments.^[^ ^41^, ^42^ ^]^ For instance, LP‐TEM has been utilized to uncover different underlying chemical/electrochemical phenomena, giving rise to the growth and nucleation of nanoparticles in inorganic materials.^[^ ^23^, ^43^, ^44^, ^45^, ^46^, ^47^ ^]^ For example, several studies have reported that Cu catalysts significantly undergo structural changes under CO_2_R conditions, impacting their catalytic activity, selectivity, and stability.^[^ ^29^, ^48^, ^49^, ^50^, ^51^, ^52^, ^53^ ^]^ Specifically, in situ TEM has been used to observe the restructuring of CuO nanosheet and Cu_2_O cube catalysts into dendritic Cu structures and fragmented nanoporous structures under electrochemical potential, respectively.^[^ ^29^, ^53^, ^54^ ^]^ Additionally, our recent work utilized in situ LP‐TEM combined with SAD to visualize the morphological and phase structure evolution of the Pd‐based catalysts under CO_2_R conditions as a function of electrode potential, identifying catalytic degradation pathways occurring in Pd/PdH_x_ catalysts under CO_2_R conditions and mechanistic reaction pathways (supported by input from density functional theory) occurring on these catalysts.^[^ ^9^ ^]^

Herein^,^ in situ LP‐TEM for electrochemical applications is extensively studied as a powerful tool for investigating various electrochemical phenomena under CO_2_R conditions.

### 1.1 In Situ Liquid Transmission Electron Microscopy for Electrochemical Applications

Technological advances in microchip‐based liquid cells for TEM have greatly contributed to the growth of the LP‐TEM research field. As a result of ongoing advancements in microfluid fabrication processes, many different reactor configurations with electron transparent windows have emerged for LP‐TEM liquid enclosure. Most commonly, two silicon nitride (SiN_x_) membranes are used for liquid encapsulation.^[^ ^55^, ^56^ ^]^ The key advantages of using membranes made of thin film SiN_x_ windows (typically 10–50 nm thick) are their robustness and the ability to prevent background scattering or absorption of electrons during imaging.^[^ ^57^ ^]^ In addition, SiN_x_ liquid cells provide advantages including high compatibility for functionalization, which allows for the integration of additional components such as resistive elements for specimen heating or electrodes for the application of electrochemical potentials.^[^ ^47^, ^56^ ^]^ Resistive elements can be integrated into the SiN_x_ microchips by depositing a thin layer on the chip's surface that is patterned to specific shapes using lithography, followed by the application of an electric current to generate resistive heating. Functional elements can be used to trigger various chemical/electrochemical reactions, providing the opportunity to expand the application of LP‐TEM to a variety of different research disciplines.^[^ ^55^, ^58^ ^]^

Using LP‐TEM microchips with integrated electrodes has enabled the investigation of numerous electrochemical processes, including the formation of solid‐electrolyte interphases in batteries,^[^ ^59^, ^60^, ^61^, ^62^ ^]^ localized corrosion reactions^[^ ^63^ ^]^ and electro‐deposition processes,^[^ ^9^, ^42^ ^]^ along with in situ electrocatalysis studies involving electrochemical water splitting^[^ ^64^, ^65^, ^66^, ^67^, ^68^ ^]^ and CO_2_R.^[^ ^9^, ^54^, ^69^ ^]^ The primary goal of in situ LP‐TEM for energy‐related applications is the ability to visually track and record time‐resolved electrochemical phenomena at the nanoscale in the presence of a liquid electrolyte.^[^ ^70^ ^]^ Williamson et al.^[^ ^43^ ^]^ were the first to report the possibility of understanding the dynamic processes of the nucleation and growth of copper during electro‐deposition via in situ LP‐TEM. The evolution of individual copper clusters at the electrode/electrolyte interface was tracked under real‐time conditions. LP‐TEM has also been utilized to study the degradation of nanostructured catalysts used in proton exchange membrane (PEM) fuel cells.^[^ ^71^ ^]^ A previous study reported the demonstration of in situ structural changes and the electrochemical behavior of Pt‐Fe nanoparticles. The nucleation/growth of the nanoparticles was highly heterogeneous and largely localized to regions of the electrode that had higher current densities in regions with higher catalyst particle loadings.^[^ ^71^ ^]^ Furthermore, in situ electrochemical LP‐TEM has been recently employed to observe electrochemical processes occurring at the anode of a lithium‐ion battery during charge/discharge cycling.^[^ ^59^, ^60^ ^]^ In these studies, the formation of dendritic solid/electrolyte interface (SEI) structures was observed before lithium electro‐deposition on the gold electrode from a battery‐grade electrolyte, where the random arrangement of the SEI layers facilitated the growth of Li dendrites. To ensure the electron beam was not convoluting the results, the electron beam dose was calibrated and controlled to be low enough to not decompose the electrolyte during the in situ electrochemical cycling measurements.^[^ ^72^ ^]^ While a major focus of in situ liquid TEM research has been on investigating the electrochemical behavior underpinning the performance of battery and fuel cell materials, the application of in situ LP‐TEM for electrochemical CO_2_R research is still in its infancy and presents some unique challenges. To date, only a few reports have utilized in situ liquid phase TEM to examine catalytically relevant materials for the electrochemical CO_2_R,^[^ ^9^, ^29^, ^50^ ^]^ a topic that will be addressed throughout this manuscript.

Despite the capabilities of in situ LP‐TEM in electrochemistry, further advances in optimization are required to select proper electrode configurations, reliably control the electrochemical potential, and track the response of electrochemically active materials to applied electrode potentials while avoiding the undesired influence of electron beam effects. Notably, keeping the electron dose rate below the threshold decomposition value of the electrolyte (i.e., the dose at which the electrolyte will decompose via radiolysis) is crucial and is a prerequisite for successful imaging under liquid environments. Control of the liquid layer thickness is also important to ensure reproducible and unprejudiced data/imaging at a low electron dose.^[^ ^13^, ^73^ ^]^ In addition, if bubble formation occurs during in situ electrochemical measurements, a pressurized flow cell could be beneficial for quick removal.^[^ ^13^, ^74^ ^]^ The consideration of optimizing the thickness of the liquid layer is discussed in detail throughout this study.

**Figure 1:**
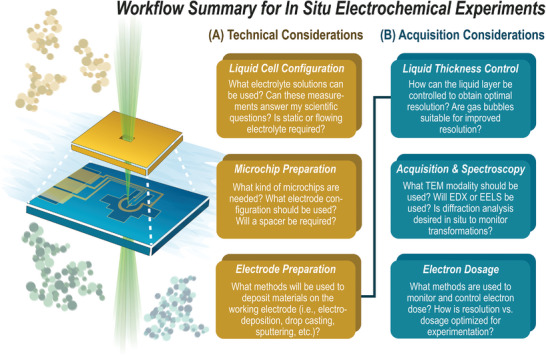
Overview of electrochemical in situ electrochemical LP‐TEM themes discussed.

There is still much to be learned and optimized for conducting in situ LP‐TEM studies of CO_2_R materials in terms of sample preparation, best practices for optimizing experimental conditions, and the data acquisition process. **Figure** **1** overviews the specific capabilities, technicalities, and implications of the in situ liquid TEM workflow for studying CO₂R electrocatalytic materials and processes. In addition, certain analytical approaches can help process the large datasets generated from in situ LP‐TEM, such as particle tracking^[^ ^75^ ^]^ and artificial intelligence/machine learning^[^ ^39^, ^76^ ^]^ analysis which can effectively monitor the evolution of electrocatalysts during CO₂ reduction. Moreover, challenges and limitations that might arise during the in situ electrochemical TEM workflow and suggested pathways to overcome such issues are summarized. To this end, workflows and experimental configurations for in situ LP‐TEM setups are suggested, demonstrated, and discussed. Moreover, a pressurized‐pump setup for flowing the electrolyte into the in situ TEM microchip is presented and compared to the use of a syringe pump. The importance of sample/electrode preparation is also highlighted, while data analysis and acquisition have been conducted to identify chemical and phase structure changes of various investigated catalysts under electrochemical operating conditions. Factors affecting the imaging resolution and quality, such as sample preparation and acquisition parameters, are discussed in detail. Finally, beam‐induced nucleation and dissolution of particles are monitored using the AXON software (Protochips Inc. Morrisville, NC, USA) to accurately measure and manage the impact of the electron beam on the sample. The goal of this paper is to highlight a systematic approach that can be taken by researchers who are interested in LP‐TEM of electrocatalyst materials and to provide tips and tricks for maximizing the efficacy and quality of their measurements. We provide a discussion on the technical considerations of performing in situ liquid TEM measurements and highlight the practical challenges that are commonly encountered. Finally, we aim to bridge the knowledge gap for non‐expert users of these powerful techniques to be able to collect meaningful data shortly after beginning their research journey.

## 2 Results and Discussion

### 2.1 Technical Requirements of the In Situ LP‐TEM Workflow: Protocols and Validation

#### 2.1.1 Liquid Cell Setup/Configuration

**Figure 2:**
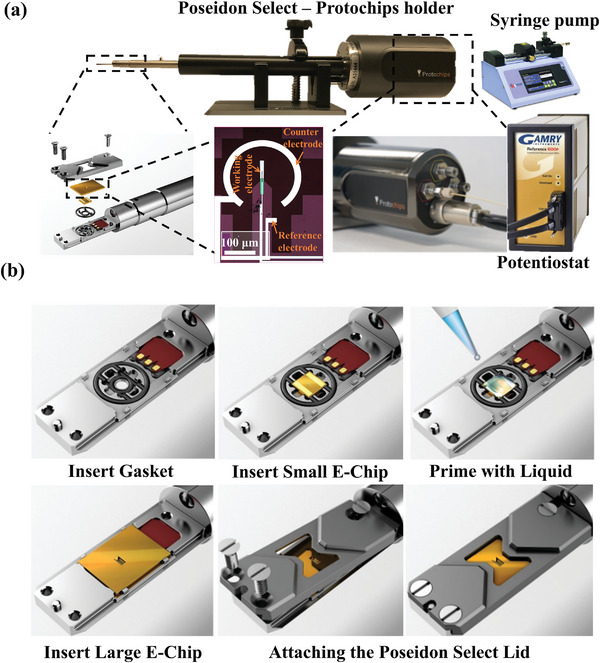
a) Protochips Poseidon Select setup for conducting in situ electrochemical LP‐TEM, incorporating SiN_x_ microchips, or E‐chips as referred to by Protochips. The E‐chips encompass a SiN_x_ membrane, whereby the bottom chip contains three electrodes (working, reference, and counter) used for in situ electrochemical measurements. b) Loading E‐chips and the assembly process.

Within this work, a commercial Poseidon Select LP‐TEM holder (Protochips Inc. Morrisville, NC, USA) is utilized, however, it is emphasized that the insights presented are widely applicable across other commercially available platforms such as Zeptools, DENSsolutions, and Hummingbird Scientific. **Figure** **2**a depicts the in situ LP‐TEM setup connected to a Gamry Reference 600+ potentiostat via a miniature banana patch adapter (MBPA) dongle. This dongle establishes a connection between the Hirose connector at the rear of the TEM holder and the Gamry cable.

The LP‐TEM holder is connected with external microfluidic tubing to a liquid electrolyte reservoir, such as a syringe for which the electrolyte flow rate can be controlled by a syringe pump. However, typical commercial syringe pumps are limited in their application to certain electrochemical processes, such as electrochemical CO_2_R. We found syringes were unable to maintain reactant CO_2_ saturation of the 0.1 m KHCO_3_ electrolyte fed to Pd‐based catalysts loaded for in situ liquid TEM. This issue was evident from the observed currents attributed to oxygen reduction that were observed in cyclic voltammetry measurements (Figure S1a, Supporting Information) when using a syringe as the electrolyte reservoir. Moreover, the pump motors of syringe pumps often contribute to mechanical oscillations which could result in pressure fluctuations and time‐variable flow rates.^[^ ^77^ ^]^ This consideration can also introduce fluctuations in the pressure and shear stress experienced within the microchip reactor that can lead to the breakage of the SiN_x_ windows or the mechanical removal of catalyst particles.

Another issue that should be considered is the pressure difference across the microchip SiN_x_ membranes (i.e., a pressure difference between the enclosed liquid and TEM column vacuum), as bowing of the membranes outward will increase liquid thickness, thus affecting the resolution of the measurements.^[^ ^41^ ^]^ Therefore, adjusting the liquid thickness and modulating the liquid electrolyte pressure under LP‐TEM conditions is necessary. To overcome the outlined limitation, the system was modified to incorporate a pressurized pump (Elvesys’ OB1 MK3, Flow controller system) setup (Figure S2, Supporting Information) wherein the gas necessary for the reaction of interest (i.e., CO_2_) was pressurized to different levels in the gas head‐space above the electrolyte reservoir. It is noteworthy that the cyclic voltammetry scan obtained using the pressurized pump demonstrated the absence of an oxygen reduction current peak (Figure S1b, Supporting Information), in contrast to the earlier observations of reduction currents attributed to oxygen reduction when a syringe was employed (Figure S1a, Supporting Information). The applied pressure was 257 mbar, resulting in a flow rate of 2.91 µL min^−1^. The pressure of the CO_2_ vapor could also be adjusted to 445.9 mbar to achieve a flow of 4.1 µL min^−1^, which provided control of the liquid flow rate while reliably keeping the electrolyte saturated with CO_2_, which is a merit of the pressure pump system. Another advantage of pressurized pump systems allow for controlling the liquid thickness in in situ LP‐TEM experiments via tuning the applied pressure, which can lead to more stability and uniformity of the liquid flow rate.^[^ ^37^ ^]^

#### 2.1.2 E‐Chip Preparation and Cleaning

Using the Poseidon Select holder, small and large SiN_x_ microchips, referred to as E‐chips, were used to encapsulate the liquid media. Before E‐chip assembly, the protective photoresist coating of the SiN_x_ windows (Figure S3, Supporting Information) must be removed by a two‐step washing process, leaving the bottom and the top surfaces with a shiny appearance. The E‐chips were first submerged in acetone for 2 min and then methanol for 2 min, followed by a gentle drying with an air duster (Figure S4, Supporting Information). Drying must be done carefully to prevent cracking the SiN_x_ membrane but strong enough to avoid solvent traces in the tip of the E‐chips. Afterward, optical microscopy was used to examine the large E‐chip at different magnifications (Figure S5, Supporting Information) to ensure that the SiN_x_ membranes were intact and that no significant particles of dust or debris had deposited onto the surface. Preceding mounting the E‐chips into the TEM holder, all E‐chips (small and large) underwent a plasma treatment for 2 min when using Pt‐based working, counter, and reference electrodes. When using a glassy carbon working electrode, only 30 s of plasma cleaning or ozone cleaning should be utilized to avoid causing damage. Plasma cleaning in our workflow was completed using a mixture of gas (nitrogen, hydrogen, and argon) under an operating power of 15 W to render the chips hydrophilic and remove any contamination (Figure S6a, Supporting Information). Afterward, a light microscope was utilized to ensure that both large and small E‐chips were not adversely affected by the plasma ions as displayed in Figures S6b,c (Supporting Information). For the large, glassy carbon working electrode E‐chip, observable damage could occur when the plasma treatment time exceeds 30 s. This was characterized by the disconnection of the glassy carbon working electrode from the Pt connector, leading to visible degradation that could potentially affect its performance under operating conditions (Figure S7, Supporting Information). In addition, the small E‐chip may be susceptible to contamination from post‐cleaning solvent residuals or exhibit defects leading to damage or breakage to the SiN_x_ window, as depicted in Figure S8 (Supporting Information). These steps involved some challenges that were overcome by strategies such as implementing meticulous cleaning protocols using high‐purity solvents and a post‐cleaning drying step to minimize the presence of residual contaminants. The proper installation for the E‐chip assembly inside the liquid TEM holder is illustrated in Figure 2b.

#### 2.1.3 Liquid Cell Assembly

As illustrated in Figure 2, a liquid sample is sandwiched between two secured E‐chips (small and large) containing SiN_x_ windows within the tip of the TEM holder for assembly. The tip of the in situ electrochemical TEM holder is a crucial component designed to facilitate precise electrochemical measurements within the transmission electron microscope. It typically consists of a specialized sample holder with an integrated electrical connection pad to enable stable electrical contact with the E‐chip that contains the sample that is to be imaged. The holder has machined pathways that allow for microfluidic electrolyte flow, with the liquid hermetically sealed in the microchip cell using a set of polymer‐based O‐rings. For this system (Figure 2), the large E‐chip has dimensions of 4 mm × 6 mm and typically comprises a 500 nm spacer. The small E‐chip has dimensions of 2 mm × 2 mm along with integrated spacers with different options for thickness including 50, 150, or 500 nm, along with the option for having no spacer. Note that for all in situ electrochemical measurements reported herein, a Gamry potentiostat with ultra‐low current capabilities (down to pico‐amperes) was used. For studying CO_2_R, the large electrochemical E‐chip was utilized which is equipped with three nano‐lithographic‐deposited electrodes, including a platinum reference and counter electrode along with either a glassy carbon or platinum working electrode.

Before loading the microchip electrochemical reactor into the TEM sample holder, the O‐ring gasket was first cleaned using sonication with distilled water and isopropanol for 10 min each, followed by drying to remove any contaminants or debris. The small E‐chip (already pre‐prepared as outlined in Section 2.2.2) was loaded onto the holder with the SiN_x_ membrane and spacer facing upward. The small E‐chip can either be inserted so that the SiN_x_ membrane is parallel or perpendicular to the length of the TEM sample holder, leading to either a parallel or cross‐configuration of the two SiN_x_ membranes (one from each E‐chip) that will be detailed in the flowing sections. To ensure that the liquid flowed smoothly between the E‐chips, a micropipette was used to dispense 2 µL of Type I Millipore water onto the small E‐chip to ensure wettability. The large E‐chip was then placed on top of the small E‐chip, ensuring that the electrode connections on the large E‐chip were in direct contact with the electronic connection pad on the TEM holder. The LP‐TEM cell was sealed and secured by tightening the small attachment screws until the top chip was flush with the tip of the TEM holder. Finally, the windows of both E‐chips were checked using an optical microscope to ensure that they were aligned so that a transparent region was observed where the two windows overlapped. Furthermore, the working electrode should be visible by optical microscopy within the transparent window of the small E‐chip. A schematic illustration of the full holder assembly is shown in Figure 2. Before inserting the sample holder into the TEM for in situ measurement, it was essential to conduct a vacuum check (Figure S9, Supporting Information) to ensure that there were no leaks or that the SiN_x_ windows would not break under vacuum conditions, which could crash the vacuum that is maintained within the TEM column. A dry pumping station was utilized to pre‐test the electrochemical E‐chips' ability to withstand vacuum conditions and to remove any residual liquid before insertion into the microscope. This procedure is crucial and aids in preventing vacuum disruption, upholding the dependability of the measurements conducted.

#### 2.1.4 Catalyst Layer Preparation on the Electrode

Several methods have been reported for precise TEM sample preparation for electrochemical studies with controlled electrode configurations. For example, focused ion beam (FIB) techniques have been utilized to prepare thin lamella samples that are deposited on E‐chips.^[^ ^78^ ^]^ However, FIB techniques have limitations, such as their destructive nature leading to the need for multiple samples or contamination, long preparation times, and high cost for sample preparation, as well as challenges when dealing with highly porous samples or samples that are prone to damage via ion‐induced ablation. Alternative methods are available for preparing electrocatalyst samples for in situ TEM imaging that can be implemented with varying levels of success. Accordingly, this section summarizes a few pathways established for preparing different types of electrocatalyst samples for in situ electrochemical TEM measurements under CO_2_R conditions, including electro‐deposition, sputtering, and drop‐casting approaches. Regardless of the method utilized for preparing samples for in situ TEM imaging, intimate electrical contact needs to be achieved between the catalyst and the working electrode patterned onto the E‐chip.

##### Electrodeposition

Electrodeposition is a convenient method to prepare catalyst layers for in situ LP‐TEM, as the E‐chips when connected to a potentiostat intrinsically provide control of electrode potentials and by extension electro‐deposition processes.^[^ ^9^, ^42^, ^79^, ^80^, ^81^ ^]^ For example, in situ LP‐TEM has been used to investigate the electro‐deposition of various transition metals including, palladium,^[^ ^9^, ^42^ ^]^ lead,^[^ ^34^ ^]^ copper,^[^ ^82^, ^83^ ^]^ and gold.^[^ ^11^, ^84^ ^]^

**Figure 3:**
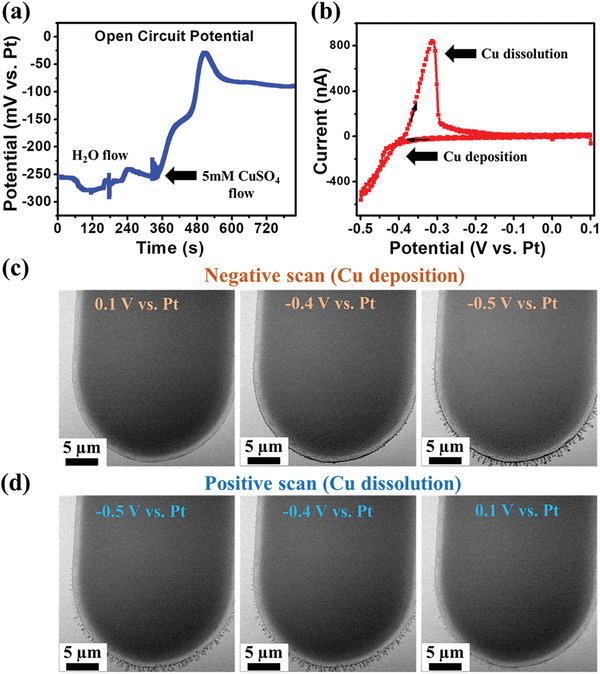
Electrochemical deposition and dissolution of Cu a) OCP tracking of the copper electro‐deposition within the first 15 min of the experiment during the time that the Millipore Type I Ultrapure water was replaced with the CuSO_4_ electrolyte solution within the in situ LP‐TEM microchip electrochemical reactor. b) Cyclic voltammetry at a scan rate of 100 mV/s and a potential window from 0.1 to −0.5 V versus Pt in 5 mm CuSO_4_ solution. c) In situ STEM images of a Pt working electrode demonstrating Cu electro‐deposition. d) Cu stripping as a function of electrode potential applied during cyclic voltammetry measurements. All images are extracted from Movie S1 (Supporting Information).

Herein, two examples are provided where electro‐deposition was used to prepare Cu particles on a Pt working electrode and Pd particles on a glassy carbon working electrode. When conducting electro‐deposition or in situ TEM measurements in general, measuring and monitoring the open circuit potential (OCP) of the working electrode can be an important diagnostic tool. First, when introducing a salt solution used for electro‐deposition, the OCP can indicate when the salt species (electro‐deposition precursors) have been effectively introduced into the electrochemical cell. For example, to confirm that the LP‐TEM reactor was filled with the CuSO_4_ electrolyte electro‐deposition solution used in this work, the open circuit potential (OCP) of the 3‐electrode configuration was continuously monitored. **Figure** **3**a illustrates the OCP tracking of the working electrode during the first 12 min that the salt solution took to displace the Millipore Type I Ultrapure water in the LP‐TEM reactor with 5 mM CuSO_4_ electrolyte solution. When Millipore Type I Ultrapure water was first introduced into the LP‐TEM reactor, the recorded OCP was ≈250 mV versus Pt, which subsequently changed to −30 mV versus Pt upon switching to CuSO_4_ solution, and eventually stabilized at −80 mV versus Pt. Before the application of any electrode potentials, a working electrode potential of 0.1 V versus Pt was applied, and under these conditions there were no particles observed on the surface of the working electrode Following this, cyclic voltammetry at a scan rate of 100 mV/s and a potential range from 0.1 to −0.5 V versus Pt in 5 mm CuSO_4_ solution (Figure 3b) was applied, with Cu electro‐deposition and dissolution observed from the resulting electrochemical response. During the cyclic voltammetry measurements, the growth and dissolution of Cu nanoparticles were observed as demonstrated in Figure 3c,d (extracted from Movie S1, Supporting Information), respectively, using STEM images captured at different electrode potentials in the negative and positive sweep directions of the cyclic voltammetry measurements under in situ conditions. Particularly, in the negative direction potential sweep, a reduction peak was observed in the cyclic voltammetry profile, beginning at −0.4 V versus Pt (Figure 3b). This reduction peak is attributed to the nucleation and growth of the electrodeposited Cu particles, which continued to grow as increasingly negative electrode potentials were applied, as shown in STEM images in Figure 3c. In contrast, during the positive direction potential sweep, an oxidation current was observed, beginning at 0.1 V versus Pt, as shown in the cyclic voltammetry profile in Figure 3b. This oxidation current corresponds to the dissolution of Cu particles depicted in STEM images (Figure 3d). It should be noted that in this study, Pt was used as a working and counter electrode for monitoring the deposition and dissolution of Cu ions. As Pt is a catalytically active metal, for example toward the hydrogen evolution reaction (HER, i.e., reductive water splitting), it is important to note that its use in liquid cells with aqueous electrolytes can introduce non‐negligible side reactions during electrochemical processes. Thus, the HER occurring on the Pt working electrode may be confounded with the CO_2_R occurring on the catalyst of interest and thus must be considered when interpreting the results of in situ experiments. Moreover, while Pt is a good catalyst for HER, it is inefficient for the oxygen evolution reaction (OER, i.e., oxidative water splitting), and therefore, the high potentials required at the counter electrode to facilitate the OER and balance the electrochemical cell can lead to Pt dissolution. This dissolved Pt can subsequently redeposit on the working electrode, which could alter surface composition and catalytic activity.^[^ ^85^, ^86^, ^87^ ^]^ Accordingly, it is crucial to take into account the roles of both the working and counter‐electrode materials to ensure accurate data interpretation. For instance, when conducting CO_2_R experiments under in situ conditions, it is recommended to use a glassy carbon working electrode instead of platinum and to perform post‐measurement ex situ analysis by techniques such as EDX to confirm that there is no influence from contamination of the working electrode by the platinum‐based counter electrode.^[^ ^9^, ^85^ ^]^

**Figure 4:**
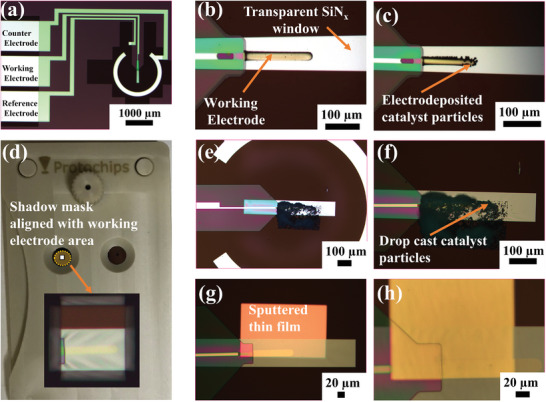
a) Light microscope images of electro‐deposited Pd on the glassy carbon working electrode prepared by applying −0.6 V versus Pt for (b) 120 s and (c) 180 s. d) Shadow mask used for sputtering and drop‐casting. e,f) Light microscope images of the drop‐cast HKUST‐1 on the glassy carbon working electrode at different magnifications. g,h) Light microscope images of the sputtered Cu thin film on the carbon working electrode with different thicknesses of 20 and 50 nm, respectively.

As another example, Pd electro‐deposition was conducted on a glassy carbon electrode containing E‐chip (**Figure** **4**a) using chronoamperometry at a fixed potential of −0.6 V versus Pt in 5 mm PdHCl_4_ with 0.015 m HCl electrolyte solution for various durations ranging from 120 to 180 s (Figure 4b,c). Electro‐deposition via chronoamperometry provides an advantage over cyclic voltammetry in that it enables tunability of the thermodynamic forces (i.e., electrode potential) driving electro‐deposition to dictate growth mechanisms and rates,^[^ ^88^ ^]^ and one can carefully control the amount of material electro‐deposited on the electrode by controlling the amount of charge (current x time) that is passed.^[^ ^42^ ^]^ When a potential of −0.6 V versus Pt was applied, a reduction current of ≈50 nA was observed (Figure S10, Supporting Information), indicating Pd deposition on the glassy carbon working electrode as confirmed by optical microscopy (Figure 4b,c).

Particularly, when the electro‐deposition time was controlled to be 120 s (Figure 4b), significantly less Pd was observed on the working electrode in comparison to 180 s (Figure 4c). Despite the useful insights that in situ TEM measurements can provide into the electro‐deposition process, differentiation between electrochemically induced and electron beam‐induced particle nucleation and growth presents challenges.^[^ ^42^ ^]^ For example, electron beam effects overall have previously been linked to rapidly increased rates of particle deposition, attributed to the generation of chemically reducing species such as hydrogen via radiolysis.^[^ ^42^ ^]^

Another challenge for in situ electro‐deposition is the electrolyte thickness, which must be high enough to enable sufficient mass transport and achieve a homogenous distribution of the catalyst on the electrode surface. For this purpose, we sought to use a liquid cell configuration with a 500 nm spacer between the small and large E‐chip, however, this corresponding increase in electrolyte thickness can affect the image resolution. To address this concern, an E‐chip cross configuration was proposed to conduct measurements close to the electrode edge with enhanced spatial resolution, as will be discussed later.

While electro‐deposition is appropriate for the preparation of working electrodes decorated with catalyst particles such as copper or palladium outlined in this section, it is not appropriate for all catalysts. For example, catalyst powders that are synthesized by alternative methods and exist in ink or slurry form are not amenable to electro‐deposition. Furthermore, owing to mass transport limitations or electric field effects, achieving conformal thin film catalyst coatings on the working electrode is not always possible. For these reasons, alternative methods for electrode preparation need to be amendable to the variety of catalyst chemistries and configurations that are of interest to the research community.

##### Drop‐Casting and Sputtering Using a Shadow Mask

In the materials and electrocatalysis community, drop‐casting or aerosol spray techniques (e.g., using a nebulizer) and sputtering approaches have become ubiquitous for uniformly depositing active layers or thin films on electrodes.^[^ ^89^, ^90^, ^91^ ^]^ Each method has its advantages and disadvantages based on the nature of the catalyst under investigation and the reaction environment. When using in situ microchip reactors, drop‐casting/aerosol spray and sputtering must address the challenge of achieving homogeneous catalyst deposition that is localized on the small (micrometer‐sized) working electrode. If localized catalyst deposition is not achieved, there is the risk of electrical short‐circuiting the electrochemical cell if catalyst particles connect the working electrode and counter (or reference) electrodes. Additionally, if regions of the catalyst‐coated electrode are too thick they may prevent proper sealing of the microchips and can lead to electrolyte leakage under vacuum conditions. Therefore, routes are needed to uniformly and locally deposit the materials of interest on the E‐chip working electrode. In this context, shadow masking can be employed to confine the deposit to the desirable area of the TEM chip.^[^ ^92^ ^]^ The shadow mask used in this work is depicted in Figure 4d, with details of the shadow mask assembly process outlined in Figure S11 (Supporting Information). The shadow mask method offers various merits such as reusability, low cost, and catalyst deposition with high micro‐meter scale precision.

Figure 4e,f demonstrates an optical microscopy image of a working electrode that has been prepared by drop‐casting a catalyst ink slurry using a commercial shadow mask (Protochips Inc. Morrisville, NC, USA). The ink slurry consisted of 1 mg of a metal‐organic framework catalyst (MOF), HKUST‐1, dispersed in 1 mL of isopropanol containing 5 µL Nafion (5 wt%). 1 µL of catalyst ink was drop‐cast onto the working electrode. The use of the shadow mask enabled localized deposition of the catalyst on the E‐chip working electrode. It should be noted that the drop‐cast catalyst layer was not completely uniform in thickness or catalyst dispersion, and it is to be understood that optimization of the ink formulation and drop‐casting method parameters is required to improve the catalyst layer quality. Particularly, we mustn't exceed a maximum catalyst particle size and catalyst layer thickness of ≈500 nm. Thicker catalyst layers may impede electron transmission and could cause the catalyst particles to break the SiN_x_ windows or prevent microchip sealing upon E‐chip assembly. For optimal performance, maintaining a catalyst particle size below 500 nm is essential. Herein, two different particle sizes of octahedral HKUST‐1 were synthesized: one at room temperature and another at 100 °C, following the methodologies detailed in previous articles.^[^ ^93^, ^94^ ^]^ Figure S12 (Supporting Information) shows STEM/EDX mapping of HKUST‐1, prepared at different temperatures (room temperature and 100 °C). Figure S12a (Supporting Information) shows HKUST‐1 particles synthesized at room temperature, exhibiting sizes less than 200 nm. In contrast, HKUST‐1 catalysts prepared at 100 °C are depicted in Figure S12b (Supporting Information), with a particle size ≈2 µm. This significant increase in the particle size, surpassing the 500 nm limit, led to the breakage of the SiN_x_ windows upon E‐chip assembly. After achieving the optimal HKUST‐1 catalyst particle size of less than 500 nm (e.g., synthesized at room temperature), the thickness of the drop‐cast catalyst layer can be managed by controlling the amount of ink deposited through the shadow mask window onto the working electrode. Successful drop‐casting is characterized by the precise localization of catalyst ink directly on the working electrode region as evidenced by the optical microscopy image shown in Figure S13a (Supporting Information). In contrast, drop‐casting using the shadow mask can be challenging when the catalyst ink is distributed around the working electrode (Figure S13b, Supporting Information). Achieving localized coverage of catalyst particles on the working electrode requires careful selection of the solvent type, ink concentration, ink deposition amounts, and the drying procedure. As the optimal conditions for drop‐casting are material‐dependent, researchers are encouraged to try out different ink formulations and drop‐casting parameters on old/used E‐chips to identify the ideal approach for their particular study.

While drop‐casting catalyst particles require optimization to control the properties of the catalyst slurry and resulting catalyst layer thickness which is fundamentally limited by the size of the largest catalyst particles, sputtering is an alternative catalyst layer fabrication technique for precise thickness control. Figure 4g,h shows optical microscope images of Cu thin films that have been prepared by sputtering on two separate E‐chips using the shadow mask, with controlled thicknesses of 20 and 50 nm, respectively, achieved by the use of a quartz crystal microbalance. Despite the advantages that sputtering offers, care must be taken to ensure that sputtering targets are pure and that the high vacuum chambers are free from contamination. Even small amounts of transition metal impurities can dramatically impact the activity/selectivity of electrochemical CO_2_R catalysts^[^ ^85^ ^]^ and likely also the structural evolutions that may occur during in situ TEM measurements. These potential issues should be addressed before time is spent attempting to conduct in situ TEM measurements on a catalyst or catalyst layers that are not amenable to the measurements, or the scientific question(s) being probed.

### 2.2 Challenges to Overcome: Data Acquisition and Analysis

**Figure 5:**
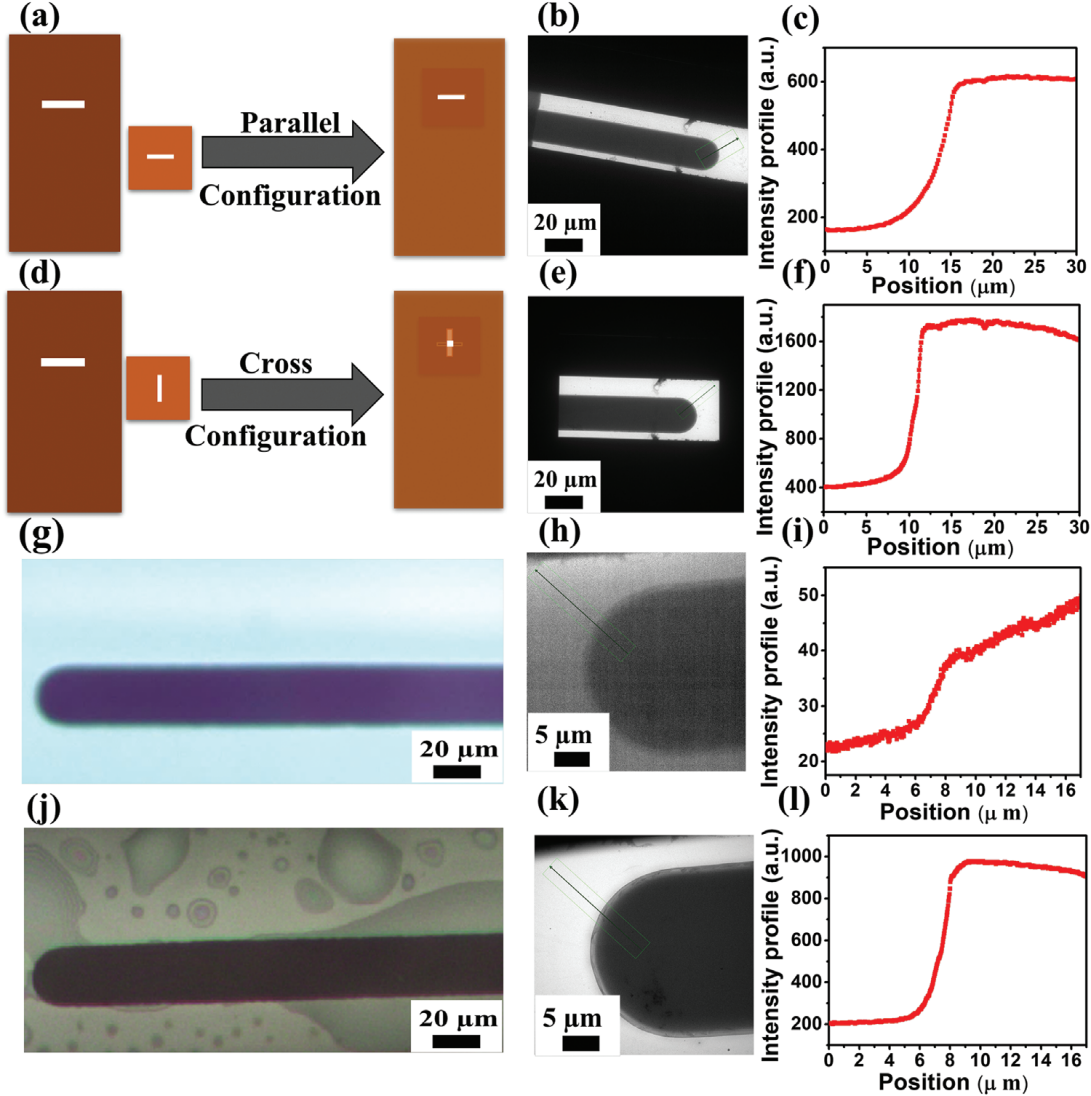
a–f) Comparison of parallel and cross configurations between the two SiN_x_ membranes, demonstrating the TEM images of both configurations with their extracted intensity profiles: a,d) Schematics of (a) parallel and (d) cross configurations. b,e) In situ TEM images of the (b) parallel and (e) cross configurations. c,f) Corresponding intensity profiles of (c) parallel and (f) cross configurations, respectively. g,j) Transmitted light microscope image: (g) before and (j) after bubble formation (extracted from Movie S2, Supporting Information). h,k) In situ TEM images of the Pt working (h) before and (k) after bubble formation (extracted from Movie S3, Supporting Information). i–l) Corresponding Intensity profiles of the in situ TEM images (i) before and (l) after bubble formation. All measurements were conducted in a CO_2_‐saturated 0.1 m KHCO_3_ electrolyte.

We turn now to a discussion of methods for controlling the thickness of the liquid electrolyte during in situ S/TEM measurements under CO_2_R conditions, using CO_2_ that has been solubilized in 0.1 m KHCO_3_ with a solubility limit of ca. 34 mm.^[^ ^95^ ^]^ From a practical standpoint, the liquid electrolyte thickness should be less than 300 nm to achieve a balance between minimizing electron absorption by the electrolyte layer to achieve high‐resolution TEM imaging,^[^ ^36^ ^]^ while maintaining an electrolyte layer that is thick enough to maintain ionic conductivity to apply electrode potentials.^[^ ^36^ ^]^ For achieving increased spatial resolution during in situ measurements, the thickness of the electrolyte can ideally be reduced to a thin film (<100 nm). One of the major advantages of decreasing the electrolyte thickness is the possibility of enhancing the contrast for better imaging and diffraction. A decreased electrolyte thickness can be achieved by using a small E‐chip with no spacer along with applying a cross‐configuration mode for the SiN_x_ windows during the in situ setup assembly to reduce the SiN_x_ bowing effect.^[^ ^22^ ^]^ Herein, two approaches for assembling the SiN_x_ E‐chips are represented and discussed, including the parallel and cross configurations, as shown in **Figure** **5**. In the parallel configuration (Figure 5a), the whole working electrode was observable within the SiN_x_ window as confirmed by the in situ TEM image shown in Figure 5b. In contrast, the cross‐configuration (Figure 5d) for the SiN_x_ windows resulted in only ≈60% of the working electrode being observable within the viewing area (Figure 5e). To provide insights into the image resolution and structural characteristics of the in situ TEM images captured under both parallel and cross configurations of the two SiN_x_ microchips, extracted intensity (brightness) profiles are introduced in Figure 5c,f,i,l. The intensity profile term mentioned here provides a quantitative representation of the variation in intensity values (electron counts) along a chosen line or path within the TEM image, offering insights into image contrast and enabling the quantitative analysis of the thickness of the electrolyte layer.^[^ ^96^, ^97^ ^]^ As shown in Figure 5c, the extracted intensity profile from a position of 30 µm from the edge of the Pt working electrode when the E‐chips were assembled in the parallel configuration revealed that the maximum intensity profile was ≈600. Conversely, in the case of the cross‐configuration setup, the maximum intensity profile extracted from the same location was 2.6x higher in magnitude (≈1600) compared to the corresponding parallel configuration (Figure 5f), indicating a substantially reduced electrolyte layer thickness. Both configurations were quantified at the same magnification and acquisition parameters using the profile length (30 µm). The cross‐configuration of the two SiN_x_ E‐chips minimized the SiN_x_ window bowing effect and minimized the electrolyte volume, thus generating a TEM image with enhanced contrast under thin‐film conditions in comparison to the parallel configuration. This difference could be attributed to the strong inelastic scattering of the electron beam that resulted in an increase in the Signal to Noise ratio (SNR) (amplitude of the signal from measured data to the amplitude of the signals from unwanted disturbances) for the TEM images captured in thin‐film conditions, resulting in more reliable data. These observations further confirmed the substantial effect of the liquid thickness on image contrast and brightness which is in agreement with previous studies.^[^ ^36^, ^98^ ^]^

#### 2.2.1 Bubble Formation During In Situ TEM Measurements under Electrochemical CO_2_R Conditions

Gas bubbles generated, via applying an electrode potential of −1 V versus Pt on the working electrode for 3 s, either in the vicinity or at the working electrode, have been shown to enable significantly improved TEM imaging capabilities.^[^ ^36^ ^]^ Under electrochemical CO_2_R conditions, gas species such as carbon monoxide, methane, ethylene, and hydrogen can be electrochemically generated which can purge most of the electrolyte out of the SiN_x_ window area, leaving behind a thin film of the liquid electrolyte covering the surface of the E‐chip, including the electrodes and cell window.^[^ ^36^ ^]^ Herein, an example of generated bubbles is presented, whereby a potential of −1 V versus Pt was applied to the Pt working electrode using the E‐chips in a cross‐configuration to generate gas bubbles under in situ CO_2_ conditions (Figure 5g–l). For the visualization of gas bubbles, an ex situ Protochips electrochemical setup, identical to the in situ TEM setup shown in Figure 2, was first utilized to visualize bubble formation using the light microscope (Figure S14, Supporting Information). Simultaneously, the light microscope was employed to observe the Pt microchip working electrode before and after bubble formation as represented in Figure 5g,j (extracted from Movie S2, Supporting Information), respectively. Figure 5h,k shows the in situ TEM images (extracted from Movie S3, Supporting Information) of the Pt working electrode before and after bubble formation, respectively, whereby the image contrast of the electrode in the presence of the bubbles was enhanced. Due to the formation of bubbles, likely consisting of hydrogen and/or carbon dioxide under the applied electrode potentials, a thin liquid electrolyte film was formed along the interior surface of the E‐chip, which significantly enhanced the imaging resolution and contrast. To empirically estimate the relative thickness of the liquid within the cell, a parameter denoted as “t over λ” was measured using Electron Energy Loss Spectroscopy (EELS) at a collection angle of (β = 30 mrad), whereby the liquid thickness within the LP‐TEM electrochemical cell was calculated to be ca. 50 nm. It should be noted that t/λ is not additive when multiple scattering occurs (when t/λ > 1) and is dependent on the collection angle (β).^[^ ^99^ ^]^ Detailed information regarding the calculations for liquid thickness can be found in the Supporting Information along with Figure S15 (Supporting Information). Furthermore, the intensity profile of both electrodes before and after bubble formation was extracted and displayed in Figure 5i,l, respectively. As can be seen, the intensity profile was initially low before bubble formation (Figure 5i) and then increased dramatically after generating bubbles (Figure 5l), resulting in a more enhanced contrast in the TEM images. It should be noted that during bubble formation the ionic conductivity throughout the three‐electrode cell must be maintained to preserve the ability to reliably apply an electrode potential to the working electrode. The ionic conductivity between the electrodes can be tracked by monitoring changes in the open circuit potential and electrochemical response such as those measured by cyclic voltammetry as previously represented in Figure 3 and Movie S1 (Supporting Information) to visualize the copper deposition and dissolution.

#### 2.2.2 Elemental Mapping in Liquid

**Figure 6:**
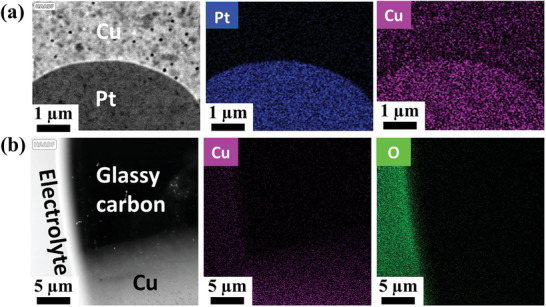
STEM/EDX mapping of the sputtered Cu measured at different areas of the E‐chip working electrode: a) Pt connector/Cu thin film interface. b) Cu thin film/electrolyte/glassy carbon electrode interface.

Identifying the chemical composition of catalysts and their elemental distribution under liquid conditions is necessary to investigate the degradation of the catalyst and electrode evolution under electrochemical CO_2_R. Therefore, S/TEM measurements are beneficial to unravel the elemental and structural transformations of different structures at a nanoscale with various elemental compositions. STEM imaging can be complemented through EDX mapping for full elemental analysis, whereby the incident electron beam in STEM mode leads to the emission of a characteristic X‐ray from the atom so that detailed elemental composition line profiles and maps can be obtained.^[^ ^100^ ^]^ Although EDX analysis within a liquid cell can be challenging due to X‐ray absorption by the membranes and liquid electrolyte, recent advancements in TEM setups, such as the inclusion of four in‐column SDD Super‐X detectors, significantly enhance X‐ray signal collection. Additionally, replacing the titanium lid of the in situ TEM holder with beryllium, which generates negligible background X‐rays, is another strategy to further improve the signal‐to‐noise ratio for low‐energy X‐ray detection.^[^ ^101^ ^]^ Under STEM mode, images are generated using high angular dark field scanning (HAADF‐STEM) to yield atomic number‐sensitive images.^[^ ^102^ ^]^ Herein, liquid layer thickness was optimized to achieve a thin film by utilizing a small E‐chip without a spacer in a cross‐configuration mode. This approach enabled achieving high‐resolution in situ STEM/EDX images through a thin layer of liquid electrolyte under CO_2_ reduction conditions. Therefore, STEM/EDX was performed to characterize the morphology of Cu thin film, synthesized by sputtering (prepared as outlined under head “Drop‐Casting and Sputtering Using a Shadow Mask”), and quantifying its elemental distribution across the electrode area in CO_2_ saturated 0.1 m KHCO_3_ at open circuit potential. As can be seen in **Figure** **6**, HAADF‐STEM‐EDX mapping demonstrated the possibility of acquiring in situ EDX maps from different areas of the glassy carbon working electrode. As shown in Figure 6a, the Pt connector (linking the glassy carbon working electrode with the three‐pad electrochemical reactor) and the Cu interface were recognized. Additionally, the interfaces of the glassy carbon working electrode with Cu and electrolyte solution are revealed in the HAADF‐STEM‐EDX shown in Figure 6b. The sputtered Cu is primarily observed on the regions of the glassy carbon working electrode where it was directly deposited, but it does not fully cover the entire surface of the electrode (Figure 4h). The contrast in the Cu map indicates a reasonable signal‐to‐noise ratio, primarily due to the reduction of the electrolyte thickness via careful sample preparation. The corresponding EDX mappings show the distribution of Pt, Cu, and O of the sputtered Cu catalysts (Figure 6), indicating that with appropriate sample preparation and electrolyte thickness control, EDX elemental mapping can be an effective tool for understanding elemental distributions in the samples being imaged. Additionally, while EELS t/λ measurements are useful for confirming the presence of electrolytes in the cell, they require significant time and effort to set up, acquire, and interpret. In contrast, EDX can quickly identify key elements in the electrolyte. For instance, Figure S16 (Supporting Information) displays the specific EDX mapping spectra associated with Figure 6a, including the Cu L, Cu K_α+β, and Pt L_α+β X‐ray emission lines in a dry cell. These X‐ray spectra do not show an oxygen peak, indicating the absence of electrolytes. In Figure S17 (Supporting Information), the oxygen peak from Area 1 shows low intensity, where thin‐film liquid is generated. Meanwhile, a stronger oxygen peak is evident from Area 2, where a more substantial liquid electrolyte surrounds the working electrode. The ratio of Si to O can also be used to estimate the amount of the electrolyte present in the cell.

EELS can be a more effective method than EDS for characterizing light elements such as Li, C, O, and N.^[^ ^109^ ^]^ However, EELS can be challenging in the presence of a liquid electrolyte, especially when the electrolyte layer is quite thick. To this end, strategies must be developed and employed to minimize the electrolyte thickness, such as the work by Serra–Maia et al. who used EELS measurements in the liquid cell to observe the K‐edge of N and O and M‐edge of Pt EELS mapping which were enabled by reducing the liquid electrolyte thickness via formation of a bubble, thus lowering the impact of electron scattering.^[^ ^36^ ^]^ On the other hand, EDS may be a more versatile tool for the liquid cell technique, particularly for heavier elements as it relies on X‐ray generation, which is less impacted by liquid thickness than electron scattering in EELS.^[^ ^103^ ^]^ While EELS provides unique insights, EDX is more straightforward for routine elemental analysis, but taken together, these techniques can be considered complementary.

#### 2.2.3 Selected Area Diffraction Analysis (SAD) for Phase Determination

**Figure 7:**
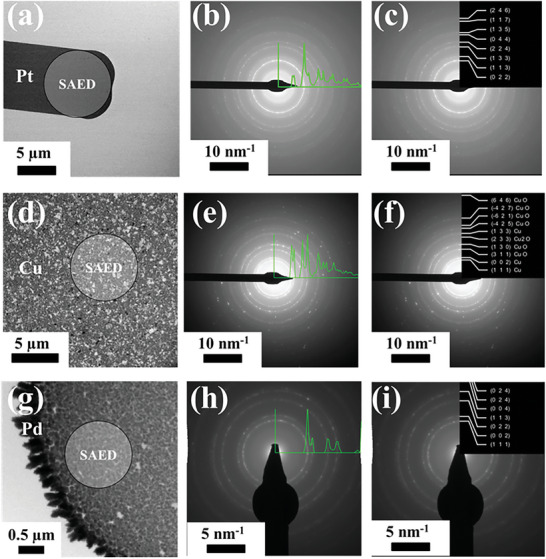
In situ LP‐TEM showing the region where SAED patterns were measured, along with the corresponding radial intensity profiles for (a‐c) Pt connector, (d‐f) Thin film sputtered Cu, and (g‐i) Electrodeposited Pd. These measurements were collected in CO_2_‐saturated 0.1 M KHCO_3_ electrolyte at a flow rate of 5 µL min^−1^ at open circuit potential (OCP).

To identify the phase structure of electrochemically active materials and correlate them to the overall reaction performance, phase identification, and unit cell determination can provide structural information in the reciprocal space. However, performing in situ electron diffraction measurements, particularly under liquid media, presents significant challenges. One of the main challenges of performing in situ electron diffraction under a liquid environment is optimizing the liquid thickness. One key aspect to address this challenge is optimizing the liquid thickness down to a thin film via bubble formation, which is advantageous under liquid media for obtaining high‐quality diffraction patterns, as reported in the literature.^[^ ^36^ ^]^ Additionally, energy‐filtered diffraction can be beneficial for filtering out diffuse scattering and isolating diffraction rings. Zero‐loss filtering in BF‐TEM or HREM can reduce diffuse scattering, improving overall image quality.^[^ ^104^, ^105^ ^]^ Moreover, internal calibration of the SAD instrumentation is of great importance to ensure reliable and accurate diffraction measurements, which represents another challenge. In this study, we developed and applied a calibration method using the Pt connector adjacent to the glassy carbon working electrode to establish a reliable reference point for in situ SAD internal calibration. SAD measurements of the Pt connector were made and calibrated to the known lattice spacing of metallic Pt to ensure that all subsequent diffraction patterns obtained are scaled correctly, contributing to the precision of the analyses. To identify and quantify the phase structures and lattice spacings, respectively, under in situ conditions, CrysTBox software was used for the crystallographic visualization and the automated analysis of the electron diffraction pattern.^[^ ^106^ ^]^ Steps for data fitting using the CrysTBox‐ring GUI are detailed in the Supporting Information (Figure S18, Supporting Information). The machine learning‐based CrysTBox tool provides the potential for identifying diffraction images/ring diffraction patterns fully automatically in a short time.^[^ ^106^ ^]^ In this work, SAD patterns were estimated and their corresponding integrated radial profiles were extracted using a Pt connector with the glassy carbon working electrode for the in situ internal calibration. The intensity radial profile method was used to calculate the d‐spacing of the investigated catalysts from in situ LP‐TEM/SAD patterns using the CrysTBox‐ring GUI and the crystallographic information file (cif) obtained from the crystal structure database. Benefitting from the facile control over the liquid thickness via bubble formation, a thin liquid layer was present which dramatically improved spatial resolution and enabled high‐quality structural analysis of catalysts by electron diffraction. This effect is demonstrated in the examples introduced in **Figure** **7**, which show the possibility of getting high signal diffraction patterns under in situ conditions. Particularly, in situ LP‐TEM and SAD (LP‐TEM/SAD) characterizations were conducted to investigate the morphology and phase structures from the Pt connector, Cu thin film sputtered on the Pt E‐chip working electrode, and Pd deposited on glassy carbon E‐chip working electrode catalysts (prepared as mentioned previously in Section 2.1.4). In situ LP‐TEM/SAD patterns with radial intensity profiles were collected in a short sequence at open circuit potential in 0.1 m KHCO_3_, where the total liquid thickness was assumed to be constant (≈100 nm). Figure 7a–c shows the in situ LP‐TEM image of the Pt connector and its corresponding SAD patterns. As can be observed in Figure 7c, all sets of planes can properly match the theoretical values stated in Table S1 (Supporting Information) for the standard metallic Pt that is set as a reference material. Moreover, Figure 7d–f substantiates the sputtered Cu thin film and its related SAD with various sets of planes including (111), (022), and (133), which can be assigned to the metallic copper, while as crystal planes of (311) and (130) could be indexed to the copper oxide phase (Figure 7f). In contrast, the copper monoxide phase could be distinguished from the corresponding reflection of the (233) plane (Figure 7f). These values are in good agreement with the theoretical values of Cu at different oxidation states, as summarized in Table S2 (Supporting Information). On the other hand, Pd‐based catalysts were reported as intriguing catalysts for study and were found to transform into a palladium hydride phase under CO_2_R conditions.^[^ ^9^ ^]^ Tracking the phase transformation of Pd to PdH_x_ under CO_2_R conditions was possible by collecting diffraction patterns of Pd‐based catalysts at several CO_2_R‐relevant electrode potentials and analyzing the data using the same automated CrysTBox tool utilized previously in our work.^[^ ^9^ ^]^ Herein, the electrodeposited metallic Pd and its SAD analysis are shown in Figure 7g–i, whereby the (111), (022), (002), and (113) planes are identified (Figure 7i), corresponding to the metallic Pd, as confirmed in our previous study.^[^ ^9^ ^]^

#### 2.2.4 Dose Rate and Cumulative Dose Effect

Dose management is an important aspect of TEM experiments due to the interaction between the sample and the electron beam energy used to form the image. A microscopist can unintentionally damage a sample which can be wasteful in terms of time and resources. Worse yet, beam damage can alter the sample's characteristics in ways that lead to erroneous conclusions about the results and raise question(s) about whether the observed reaction is a result of the applied stimulus, beam damage, or a combination of both.^[^ ^107^ ^]^ Consequently, low‐dose imaging techniques have been developed to mitigate the damaging effects of electron/sample interactions.^[^ ^47^, ^108^, ^109^ ^]^ For instance, Integrated Differential Phase Contrast Scanning Transmission Electron Microscopy (iDPC‐STEM) and 4D Scanning Transmission Electron Microscopy (4D‐STEM) are effective techniques used to image beam‐sensitive crystalline materials, particularly those containing low‐Z elements such as C, N, O, and H that are commonly difficult to image when present alongside heavier elements. These techniques have been successfully applied to materials like metal‐organic frameworks (MOFs) and hybrid halide perovskites, allowing imaging at ultra‐low electron doses ranging between 5 and 30 e Å^−^^2^s^−^^2^ and cumulative doses of <100 e Å^−^^2^.^[^ ^110^, ^111^, ^112^ ^]^ Determining the electron dose is critical to preserving beam‐sensitive materials and ensuring data accuracy. Several methods can be used to measure the dose during in situ experiments. For example, an integrated Faraday cup connected to a current meter with picoampere sensitivity in the TEM can directly measure the probe current. By knowing the exposure time, an area exposed to the electron beam, and the elementary charge, the electron dose can be estimated. Additionally, a fluorescent screen within the microscope can offer an indirect means of measuring the electron dose, which is proportional to the electron exposure readout and typically based on luminescence intensity. However, the accuracy of this method is limited due to secondary electron generation within the phosphorous screen. Alternatively, an electron‐counting direct detecting camera on an EELS spectrometer can be used to count electrons with minimal readout noise, enabling imaging micrographs at very low electron doses.^[^ ^113^, ^114^, ^115^ ^]^

However, managing beam effects during TEM measurements has been limited by several issues including the difficulty of accurately calibrating TEM electron beam current and area as well as the need to maintain constant dose rates below damage thresholds. In addition, the need to manually determine a sample's dose threshold is problematic.^[^ ^107^ ^]^ To address such issues, a machine‐vision tool named AXON dose has been designed and is commercially available from Protochips Inc. This tool enhances the efficiency of such experiments by individually monitoring and adjusting the dose rates on a per‐pixel basis, thus compensating for any potential sample drift and mechanical errors.^[^ ^97^ ^]^

To incorporate beam dose considerations into in situ TEM workflows, it is recommended to initiate the process with a calibration of the beam current. Following this, the beam area is measured before any in situ assessments are carried out. This is made possible with the help of a Faraday cup linked to a pico‐ammeter which can fully collect and accurately measure the current, thus facilitating the calibration process. An example is the Protochips Dose holder, which is designed to support a workflow that measures both current and area.^[^ ^97^ ^]^ This design incorporates a Faraday cup for precise current collection, and a through‐hole enabling the measurement of beam area through the camera (Figure S19, Supporting Information).^[^ ^97^ ^]^ When combined with AXON software control, these fiducials resulted in an automated calibration process.^[^ ^97^ ^]^ Having characterized beam current, a precise calculation of the electron dose rate (e Å^−2^s^−^^2^), as well as the cumulative dose (e Å^−2^) for each pixel, can be measured.^[^ ^97^ ^]^

It should be noted that there are terminology inconsistencies in the in situ TEM literature about “electron flux density” and “electron dose rate”. Electron flux density, expressed in electrons per unit area and time, describes the number of electrons reaching the detector, and is typically what is quantified and reported during TEM measurements,^[^ ^114^ ^]^ but it should be noted that this is commonly (misleadingly) referred to as electron dose. In contrast, the electron dose rate, in Gray per second, measures the radiation absorbed by the specimen due to inelastic scattering, which is essential for assessing material resistance to irradiation. While it is theoretically possible to convert between electron flux and electron dose, this is non‐trivial because specific values for stopping power, inelastic mean free path, and sample thickness are required.^[^ ^116^ ^]^ In this paper and most reports of in situ TEM studies, the electron dose rate is reported as e Å^−^^2^s^−^^2^ based on measurements from microscope fluorescent screen screens or measuring the probe current with a picoampere Faraday cup apparatus installed in the tip of a holder or within the microscope itself. We will use the terminology “electron dose” throughout this manuscript to refer to what is an electron flux to be consistent with the literature in the field and with the terminology used by electron microscope manufacturers. Readers are encouraged to be aware of these fundamental differences and perhaps the field as a whole can work toward standardization of terminology.

**Figure 8:**
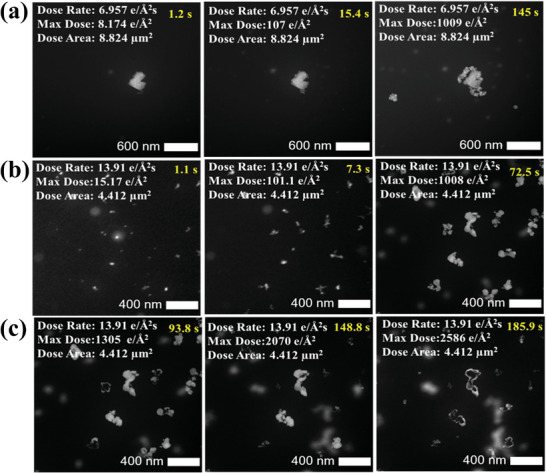
STEM images illustrate beam‐induced nucleation and dissolution of Ag‐Zn particles synthesized in a mixture of 5 mm silver nitrate and zinc sulfate monohydrate electrolyte solution and imaged in a Poseidon liquid cell under electron beam irradiation using different dose rates of: a) 6.9 e^−^ Å^−2^s^−^^2^ extracted from Movie S4 (Supporting Information). b,c) 13.91 e^−^ Å^−2^s^−^^2^ extracted from Movie S5 (Supporting Information) at different cumulative dose rates across a sample area. Note that the acquisition frame rate is 1 frame per second, and the maximum dose is calculated as the product of the dose rate and the exposure time (in seconds).

It is noted that specific threshold values for electron dose and accumulative dose rates can vary due to several factors such as the type of material being tested, the electron microscope used, and the operating conditions. Thus, precise quantification of electron dose and dose rate thresholds throughout the experiment is particularly important to minimize the impact of beam/sample interaction to a level where its impact on the results is negligible.^[^ ^97^, ^107^ ^]^ For instance, exceeding the dose threshold for ceramic and metallic samples can induce the formation of crystal defects within the sample.^[^ ^97^, ^107^ ^]^ Other samples, like zeolite specimens and biological catalysts, can be damaged at an accumulative dose of ≈100 e Å^−2^^[^ ^117^ ^]^ It is noteworthy that under liquid cell transmission electron microscopy conditions, beam interaction through aqueous solutions causes radiolitic decomposition of the liquid electrolyte into reducing and oxidizing species, causing observable changes in the material. This phenomenon can, for example, drive the nucleation and growth of metallic nanoparticles.^[^ ^97^ ^]^ To further demonstrate the importance of electron dose rate under in situ liquid conditions, the growth and dissolution of Ag‐Zn particles by the electron beam were explored, with a dose tracked using the AXON Dose tool. STEM micrographs imaged in a Poseidon liquid cell are shown in Figure 8 after a mixture of silver nitrate and zinc sulfate monohydrate electrolyte solutions were pumped into the liquid flow in situ TEM setup. As can be observed in **Figure** 8, along with Movies S4,S5 (Supporting Information), a sudden change in the particle shape‐ induced by the dose applied during the imaging process was observed along with the growth and dissolution of particles. Figure 8a shows qualitatively the particle growth with time as a factor of the dose rate. At a lower dose rate of 6.957 e Å^−^^2^s^−^^2^, nucleation, and particle growth happen gradually over 145 s, with the accumulated dose rising from 8.174 to 1009 e Å^−^^2^. In contrast, when the dose rate is increased to 13.91 e Å^−^^2^s^−^^2^, particle growth occurs much faster, completing in just 72.5 s with an accumulated dose of 1008 e Å^−^^2^ (Figure 8b). However, after 93.8 s at this higher dose rate, particle dissolution begins, and by 185.9 s, complete dissolution is observed (Figure 8c). This dose‐dependent behavior in liquid‐cell TEM is typically driven by radiolysis, which alters the local chemical environment by producing a reducing (e_h_^−^) and oxidizing (OH^•^ ) agent. Increased dose rates shift the concentration ratio of e_h_^−^ and OH^•^ which can affect the local pH and the balance between deposition and dissolution.^[^ ^86^ ^]^ To avoid beam‐induced deposition or dissolution of Ag‐Zn particles in the liquid, it is recommended to keep the dose rate below 6.957 e Å^−^^2^s^−^^2^ and the total accumulated dose below 1000 e Å^−^^2^ for this system. This advanced technology presents new possibilities for imaging samples that are sensitive to dose and also leads to further insights into the development of more advanced machine‐vision capabilities in the future.^[^ ^107^ ^]^

## 3 Summary

In situ electrochemical LP‐TEM is a powerful technique for characterizing the morphology and properties of electrocatalyst materials, enabling real‐time tracking of their dynamic changes in liquid environments under relevant reaction conditions with high spatial and temporal resolution. However, LP‐TEM measurements face challenges, such as controlling the liquid thickness, beam‐induced effects, and difficulty in maintaining sufficient image resolution in a liquid environment, all of which render LP‐TEM a non‐trivial technique. Of particular benefit to those currently involved or interested in these types of experiments is a summary of suggested approaches and best practices for performing in situ electrochemical LP‐TEM measurements and the interpretation of collected results.

Herein, a condensed technical background about in situ liquid TEM as well as a discussion of the practical aspects of workflow development are presented. Specific sets of experimental considerations for conducting in situ LP‐TEM electrochemical measurements are highlighted to achieve conditions that mimic, as closely as possible, the local reaction environments that electrocatalysts are exposed to in operating electrochemical devices. A particular emphasis herein is placed on electrochemical CO_2_ conversion. First, a discussion of the various techniques for catalyst layer/electrode preparation for in situ LP‐TEM measurements is provided, including electro‐deposition, sputtering, and drop‐casting, with the advantages and limitations of each method presented. An overview of the challenges facing in situ LP‐TEM measurements is presented, alongside a discussion of suggested best practices for addressing these challenges. A particularly critical challenge in performing electrochemical in situ LP‐TEM studies is maintaining a thin liquid electrolyte layer to provide sufficient contrast and spatial resolution during electrocatalyst imaging, whereby strategies to address this challenge are identified. Particularly, these strategies include using a small E‐chip with no spacer, along with applying a cross‐configuration mode for the SiN_x_ windows during the in situ setup assembly. Moreover, within the methodological approach reported here, generating and entrapping gas bubbles under in situ electrochemical CO_2_R conditions between the SiN_x_ viewing windows was demonstrated as beneficial to enabling improved spatial resolution, while maintaining a thin electrolyte layer over the working electrode surface that enables ionic conductivity between the three electrodes (working, reference and counter electrodes). Another significant consideration during in situ LP‐TEM measurements is the possibility of radiation damage and undesirable beam‐induced effects that can emerge at the expense of achieving high spatial resolution with increased electron doses. We highlight the need to determine radiation thresholds that should not be exceeded during in situ LP‐TEM measurements to ensure that the morphology and property changes observed in the electrocatalyst materials are due to the applied electrochemical stimulus with minimal or ideally no electron beam‐induced artifacts. Determining radiation thresholds can be achieved by precisely quantifying the electron dose using various tools, including hardware such as a Faraday cup or software tools provided by commercial suppliers of in situ LP‐TEM setups (in this case the Axon Dose tool provided by Protochips). Ultimately, the goal of this manuscript is to provide a discussion of the important challenges and considerations of in situ LP‐TEM measurements, alongside a suggested account of best practices and strategies evidenced through experimental case studies to reduce the barriers for new (and experienced) researchers to apply in situ LP‐TEM measurements for answering their pertinent research questions. We place a focus on electrochemical CO_2_ conversion electrocatalyst studies, however, it is expected that the discussions and strategies outlined in this article will be useful to researchers working on a variety of materials science challenges facing electrochemical energy conversion and storage technologies.

## Conflict of Interest

The authors declare no conflict of interest.

## Author Contributions

A.M.A. and K.E.S. contributed equally to this work. A.M.A. and D.H. conceptualized the idea and designed the methodology for the study. D.H. edited the writing and revised the manuscript. D.H. performed the funding acquisitions and supervision. A.M.A. and K.E.S. performed data curation and formal analysis. K.E.S. and A.M.A. wrote the original draft of the manuscript. A.M.A. and K.E.S. reviewed and edited the manuscript. L.‐A.D. helped with discussion and data interpretation, reviewed and edited the manuscript. F.I. synthesized HKUST‐1 catalysts and reviewed the manuscript. R.A. prepared sputtered Cu catalysts and reviewed the manuscript. K.G. reviewed the manuscript and provided scientific guidance.

## Supporting information



Supporting Information

Supplemental Movie 1

Supplemental Movie 2

Supplemental Movie 3

Supplemental Movie 4

Supplemental Movie 5

## Data Availability

The data that support the findings of this study are available from the corresponding author upon reasonable request.
