# Supplementary material for: In Situ Transmission Electron Microscopy of Electrocatalyst Materials: Proposed Workflows, Technical Advances, Challenges, and Lessons Learned
Source: Small Methods. 2024 Dec 20;9(1):2400851. doi: 10.1002/smtd.202400851 (PMC11740959; doi:10.1002/smtd.202400851)
Supplement: Supplementary file 1 — Supporting Information [file SMTD-9-2400851-s005.pdf]

small
methods

Supporting Information

for *Small Methods*, DOI 10.1002/smtd.202400851

In Situ Transmission Electron Microscopy of Electrocatalyst Materials: Proposed Workflows, Technical Advances, Challenges, and Lessons Learned

*Ahmed M. Abdellah, Kholoud E. Salem, Liza-Anastasia DiCecco, Fatma Ismail, Amirhossein Rakhsha, Kathryn Grandfield and Drew Higgins**

Supporting Information Data

In Situ Transmission Electron Microscopy of Electrocatalyst Materials: Proposed Workflows, Technical Advances, Challenges, and Lessons Learned

Ahmed M. Abdellah [1,2], Kholoud E. Salem [1], Liza-Anastasia DiCecco [3,4], Fatma Ismail [1], Amirhossein Rakhsha [1], Kathryn Grandfield [3,5], Drew Higgins [1,2*]

[1]Department of Chemical Engineering, McMaster University, Hamilton, ON, L8S 4L7, Canada

[2]Canadian Centre for Electron Microscopy, McMaster University, Hamilton, ON, L8S 4M1, Canada

[3]Department of Materials Science and Engineering, McMaster University, Hamilton, ON, L8S 4L8, Canada

[4]Department of Biomedical Engineering, The Pennsylvania State University, University Park, PA, 16802, USA

[5]School of Biomedical Engineering, McMaster University, Hamilton, ON, L8S 4L7, Canada

Email: higgid2@mcmaster.ca

A.M.A. and K.E.S. contributed equally to this work.

Keywords

electrocatalysis, electrochemical CO_2 conversion, in situ characterization, in situ liquid-phase transmission electron microscopy, operando characterization, workflow

A) Experimental Section

E-chip electrode preparation under electrochemical using in situ LP-TEM setup:

i. Pd particle electrodeposition under in situ LP-TEM environment

This research follows the microchip electrochemical cell assembly mentioned in detail in our previous study.[1] In short, a liquid solution of 5 mM H_2 $PdCl_4$ with 0.015 M HCl was introduced at a flow rate of 5 μL/min through the microfluidic channels of the sample Poseidon Select holder using an external syringe pump. Once the solution was introduced, electrochemical chronoamperometry was carried out at 0.2 V vs. RHE using a floating potentiostat (Gamry Reference 600+) for 120 and 180 seconds to compare and ensure the electrodeposition of a sufficient amount of Pd particles on the working electrode. Following electrodeposition, the in situ TEM holder was purged with Millipore water for further cleaning to remove the electrodeposition solution.

ii. HKUST drop-casting solution

Two different particle sizes of octahedral HKUST-1 were synthesized: one at room temperature and another at 100°C, following the methodologies detailed in previous articles.[2, 3]

That was synthesized by reacting Cu nitrate ($Cu(NO_3)_2$, Sigma-Aldrich) and trimesic acid ($C_9H_6O_6$, ben- zene-1,3,5-tricarboxylic acid, Sigma-Aldrich). 1.82g Cu nitrate was dissolved in 50 mL methanol while 0.875g trimesic acid was dissolved in 50 mL methanol. After vortexing to get a homogeneous solution, the Cu nitrate solution was transferred to the trimesic acid solution. The mixed solution was stirred for 2 h at room temperature and 100°C. Following stirring, a solution containing HKUST-1 was subsequently washed by centrifugation with methanol and vacuum-dried.

iii. Cu thin film sputtering on the E-chip working electrode

Copper deposition took place using a 99.999% purity copper target (LTS Chemical Inc., Chestnut Ridge, New York) with a Torr Compact Research Coater sputtering system (New Windsor, New York). A DC of 45 A was used to create the argon (> 99.999% purity, AlphaGaz, Air Liquide) plasma with the argon flow rate of 8 sccm. The deposition thickness was monitored via a quartz crystal sensor with a rate of 0.8 Å/s.

B) Figure List (Figure S1 to S10)

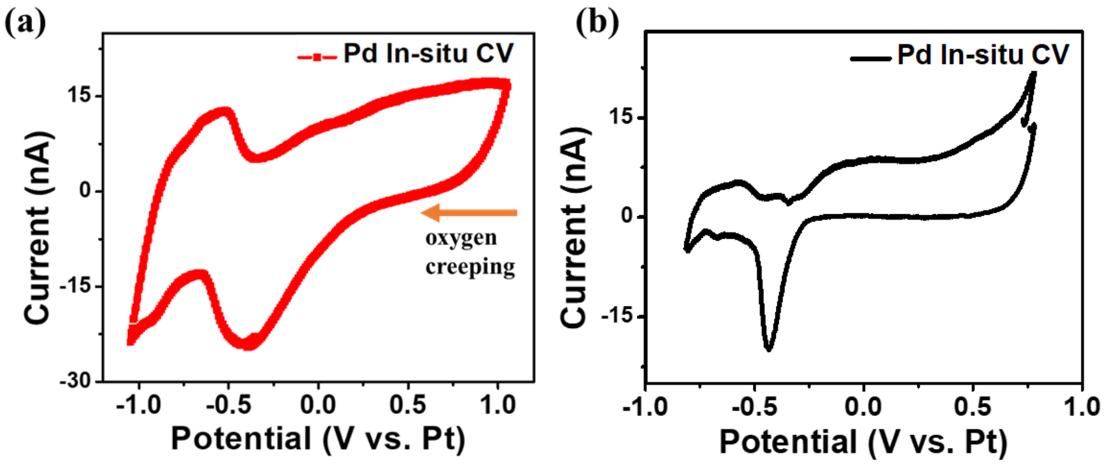

Figure S1. Cyclic voltammetry scan of Pd electrode in CO_2 saturated 0.1M $KHCO_3$ at a scan rate of 100 mV/s with measurements done using a: (a) Syringe pump, leading to a broad reduction peak attributed to both Pd reduction and the oxygen reduction reaction arising from ambient oxygen/air creeping into the electrolyte *via* the glass plunger in the syringe that contains the reactant CO_2 and electrolyte. (b) Pressure pump operating at 445.9 mbar, leading to only a sharp reduction peak due to Pd reduction and indicating an absence of any oxygen/air creeping into the electrolyte reservoir.

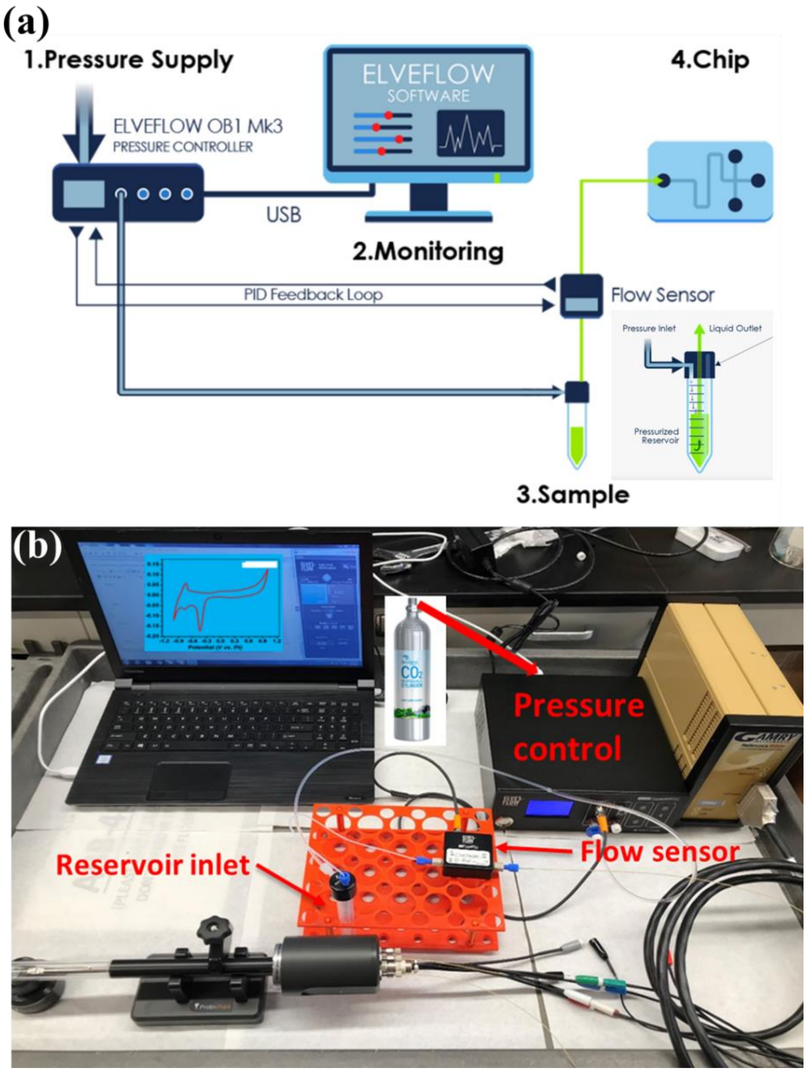

Figure S2. (a) Schematic representation of the **Elvesys' OB1 MK3 Flow Controller System**, a pressurized-pump setup. (b) Configuration of the pressurized-pump setup for in situ LP-TEM experiments. A CO_2 gas cylinder serves as the pressurized gas source and is connected to a pressure controller, which regulates the pressure applied to push the electrolyte from the reservoir through the flow sensor, maintaining a controlled flow rate of 5 μL/min inside the in situ TEM holder. Please note that while recent innovations from commercial suppliers of in situ TEM holders can enable simultaneous control of temperate and electrochemical potentials, in our setup, we do not have the ability to control the temperature, so all experiments were conducted at room temperature. Another challenge is maintaining a stable pH during reactions that either produce or consume

OH^-/H^+ ions. This can be managed by continuously flowing the electrolyte or operating at lower reaction rates to minimize drastic pH shifts.

Pressure difference calculation in the pressurized pump setup:

The pressure differential (ΔP) of the pressurized pump is calculated as the difference between the outlet pressure (P_{out})- (P_{in}):

$\Delta P = P_{out} - P_{in}$

This pressure differential (ΔP) can also be expressed in terms of the flow rate (Q) and microfluidic resistance (R) using the equation:

$\Delta P = Q \times R$

Where:

- ΔP: Pressure differential
- Q : Flow rate (5μL/min)

Using this formula, the pressure generated by the pressurized pump can be precisely calculated.

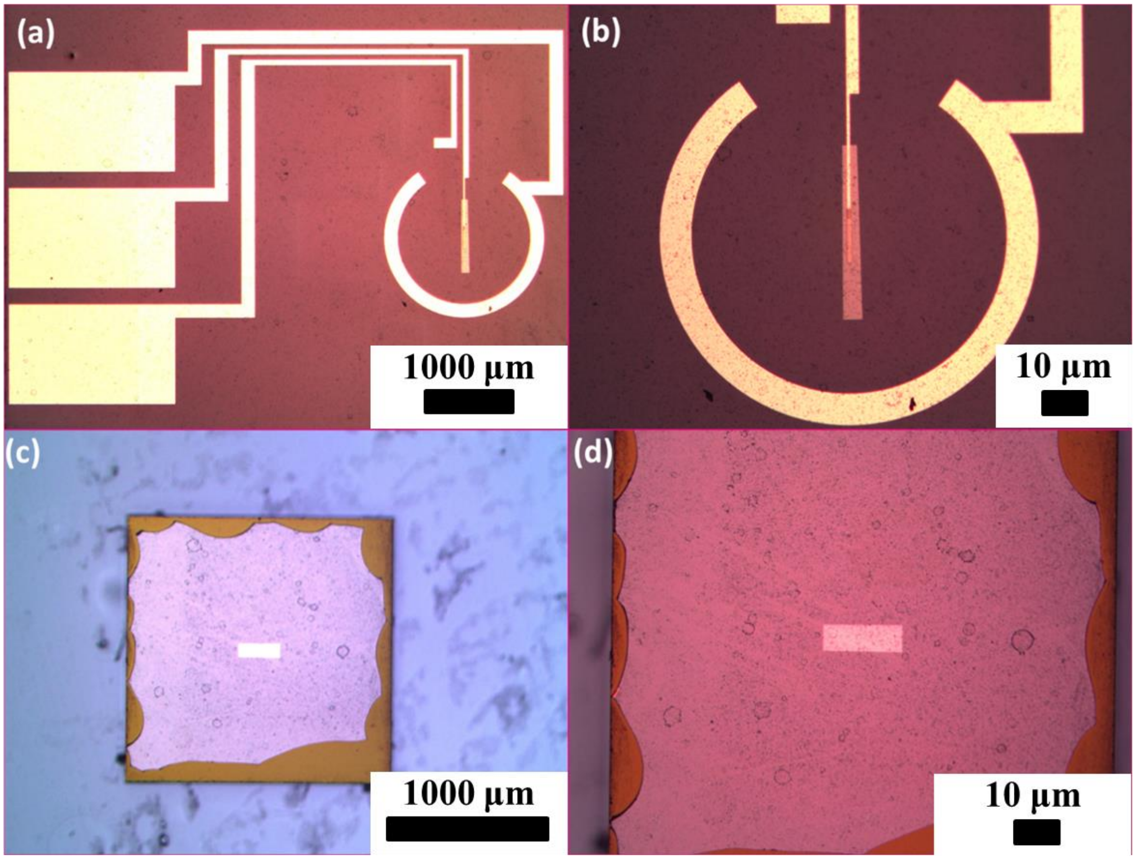

Figure S3. (a) E-chips Light microscope images with protective photoresist coating. (b) Large E-chip electrode. (c) and (d) Small E-chip shown at different magnifications.

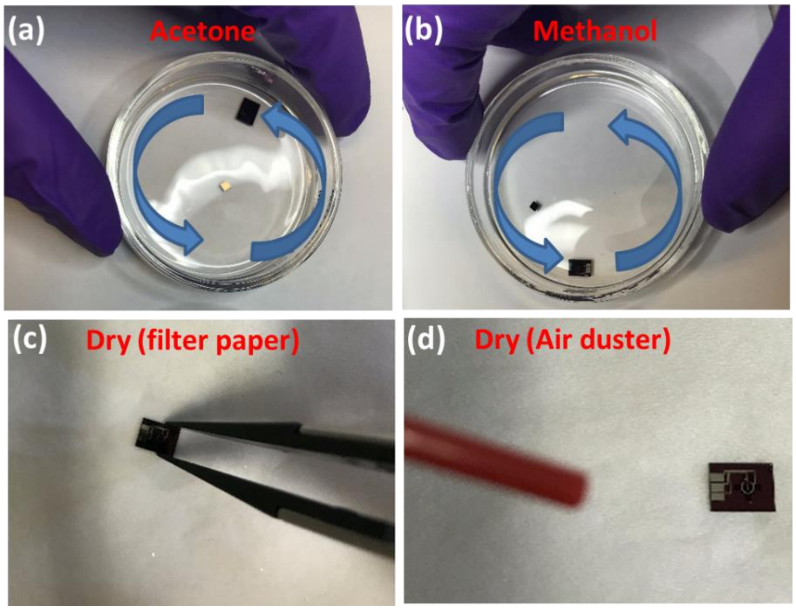

Figure S4. Steps for removing the protective photoresist layer: (a) Rinsing in acetone, followed by (b) A methanol rinse, then (c) and (d) Drying using filter paper and an air duster, respectively.

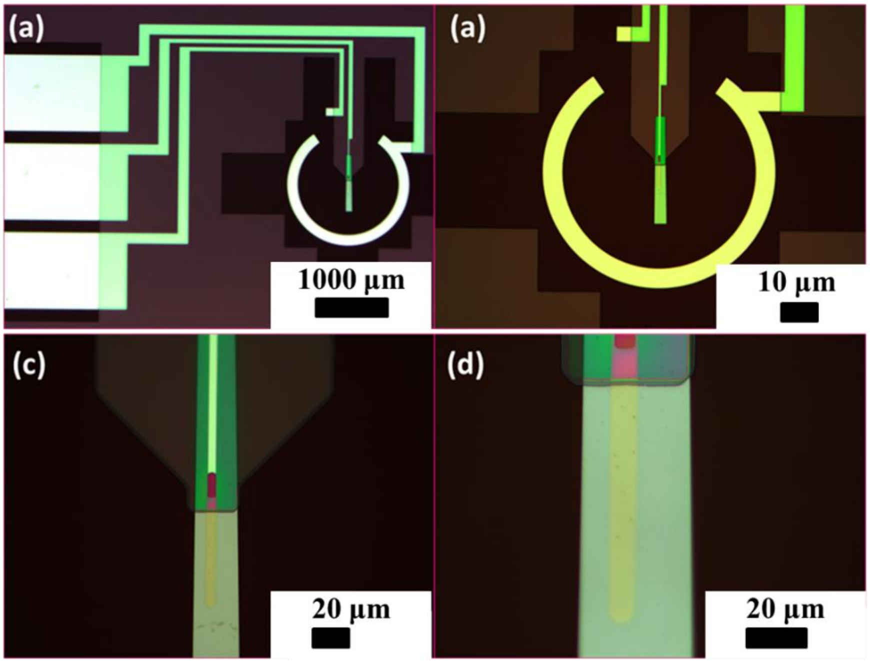

Figure S5. E-chips Light microscope images after removing protective photoresist coating: (a) and (b) Low magnification images of the large E-chip with the three electrodes (Pt working, counter, and reference electrodes) at 1000 µm and 10 µm, respectively. (c) and (d) High magnification images of the Pt working electrode captured at different regions.

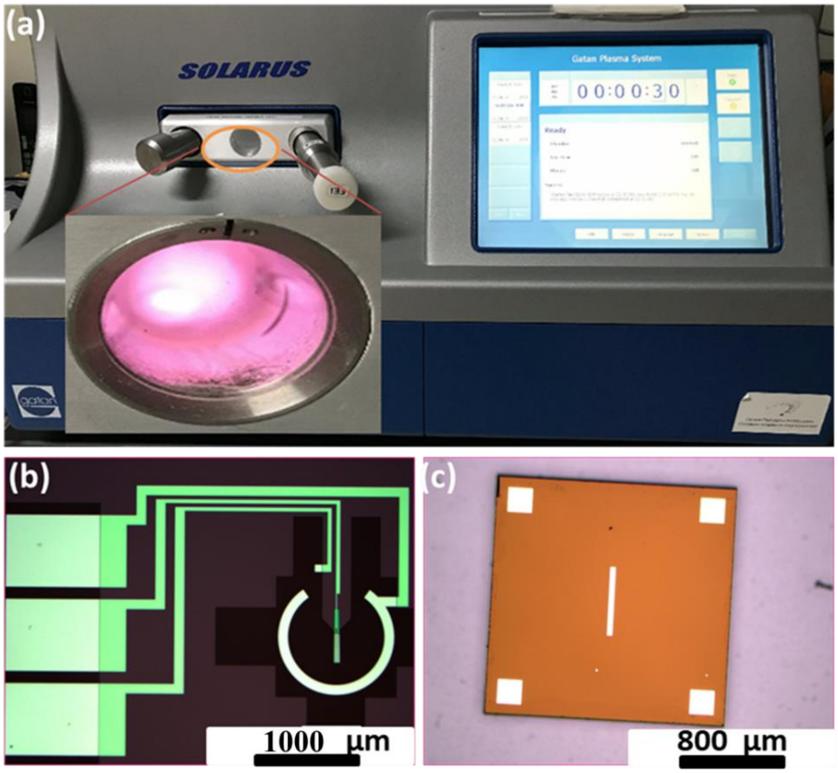

Figure S1. (a) Plasma cleaning setup, Light microscope images of the non-damaged: (b) Large E-chip. (c) Small E-chip after the plasma cleaning process.

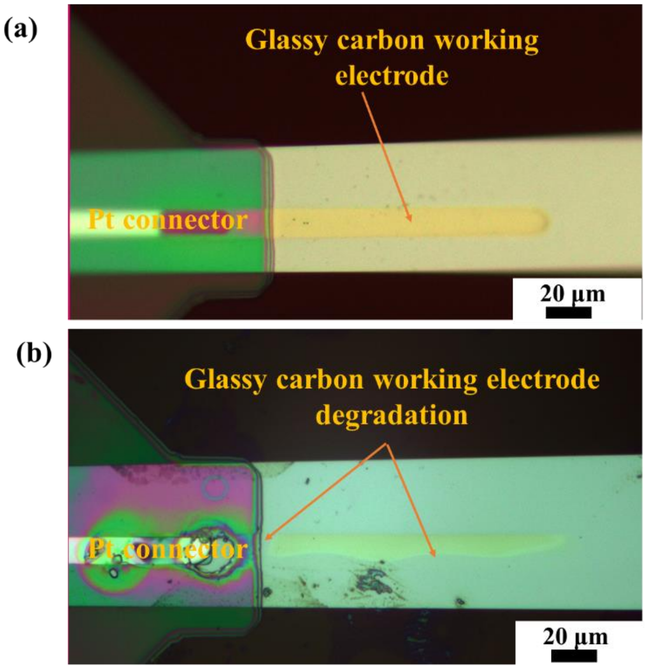

Figure S7. Light microscope images of the glassy carbon working electrode: (a) Before and (b) after plasma treatment. Note that after 30 seconds of plasma cleaning for the glassy carbon E-chip working electrode, there is observable degradation and disconnection from the Pt connector.

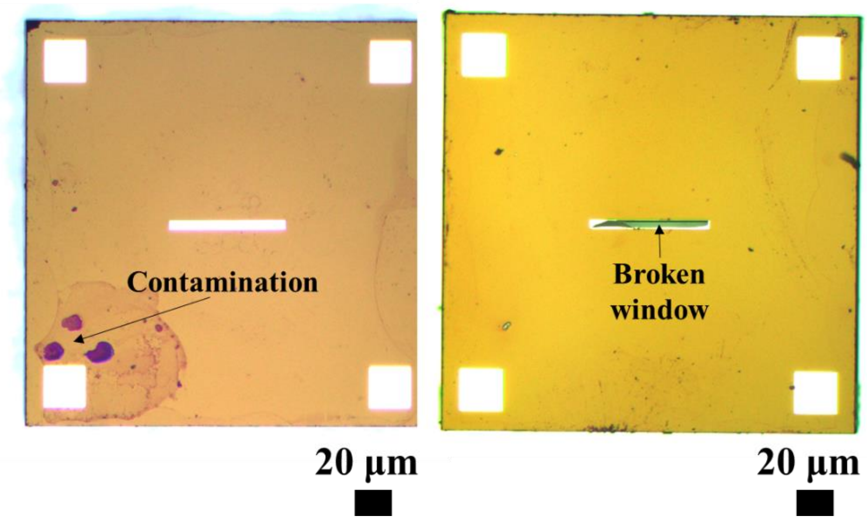

Figure S8. Light microscope images of the small E-chips showing contamination and a broken SiN_x window.

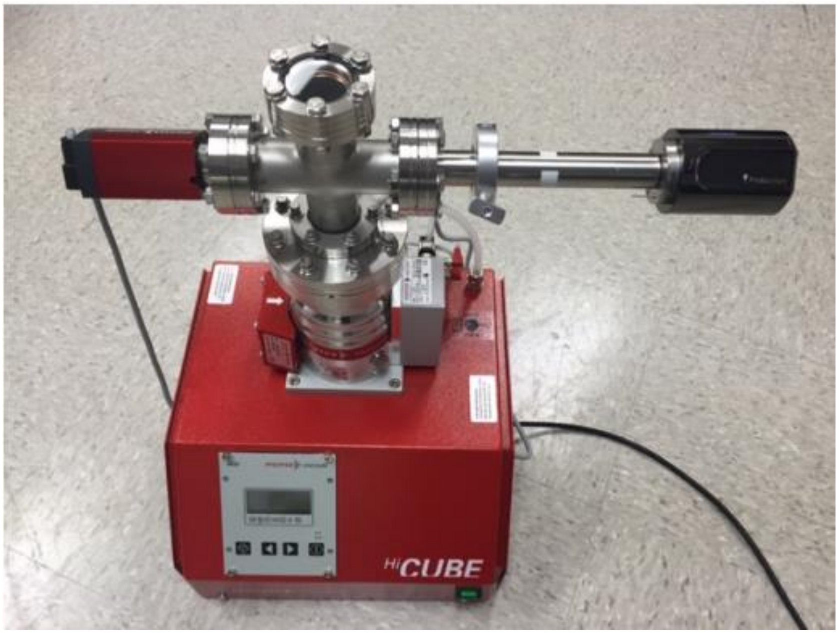

Figure S9. A vacuum check setup is used to check the holder vacuum and remove any residual liquid from loading before insertion into the microscope. The leak check is passed if it attains a vacuum level of approximately 5.5 x 10^{-6} mbar or lower.

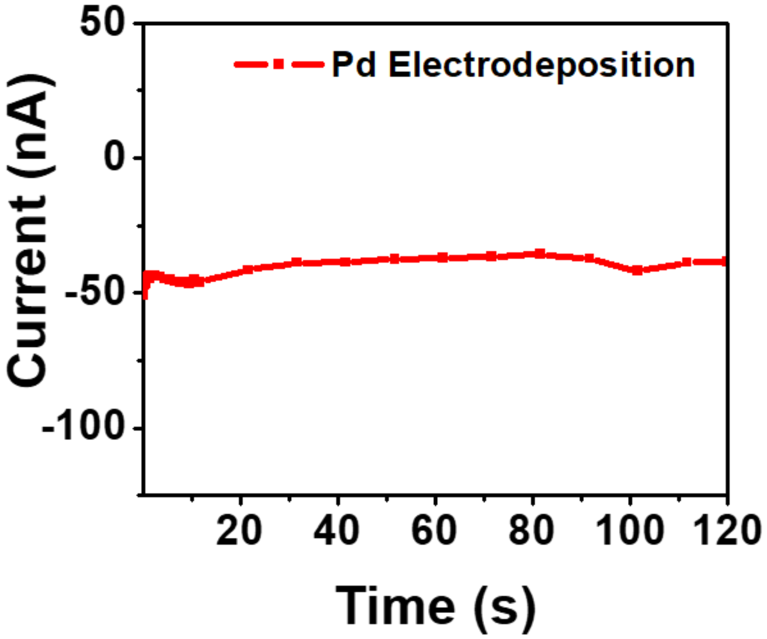

Figure S10. Palladium Electrodeposition prepared by in situ chronoamperometry at -0.6 V vs. Pt for 120 sec, in 5 mM H_2 $PdCl_4$ with 0.015 M HCl.

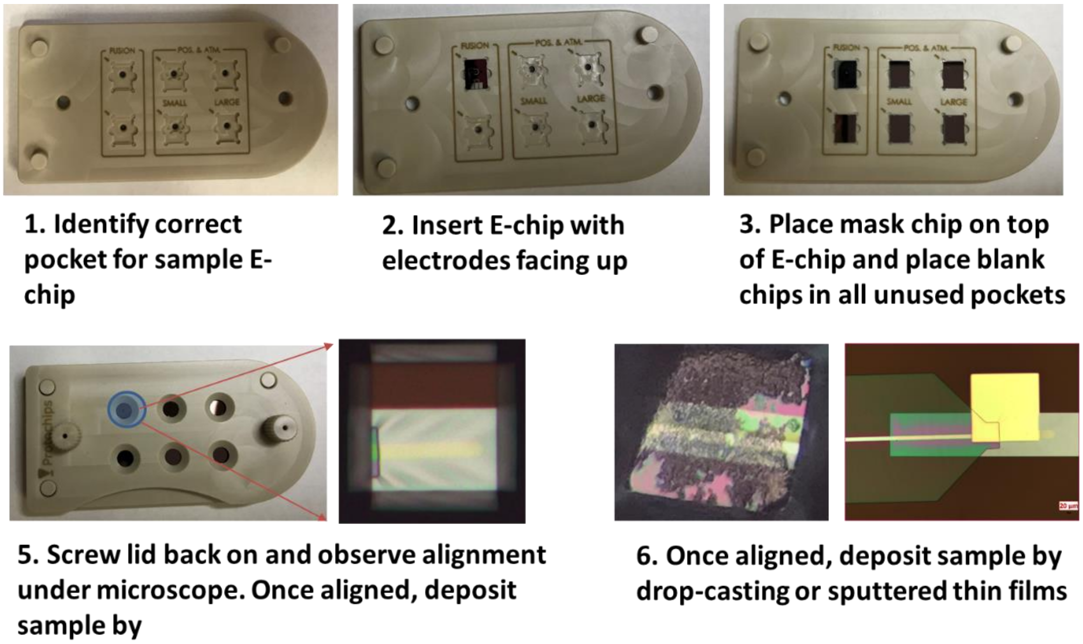

Figure S11. Steps for the loading and assembly process of the microchips using the shadow mask.

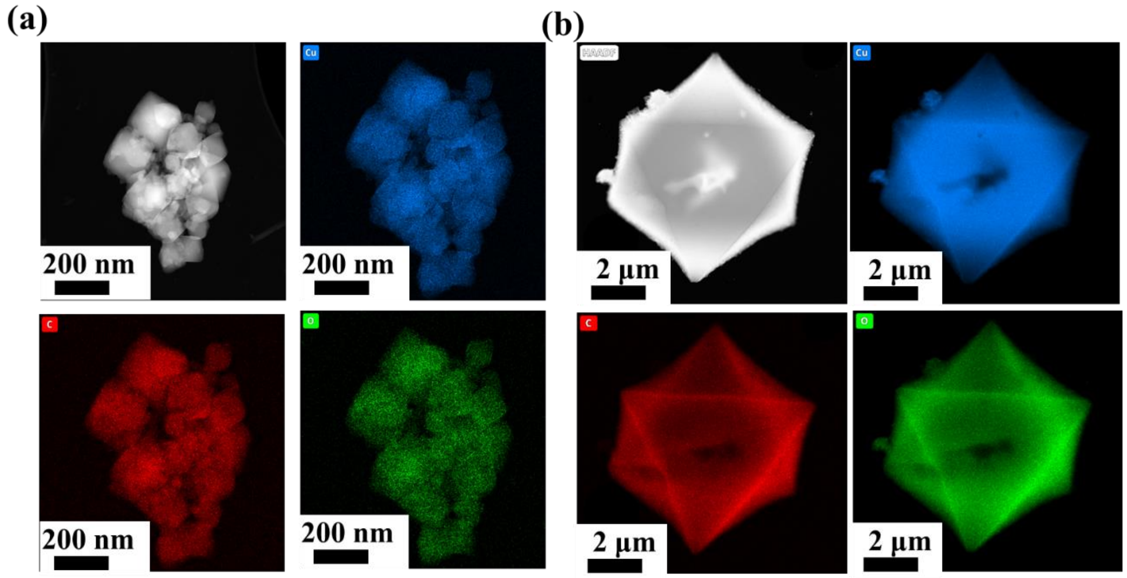

Figure S12. STEM/EDX mapping of HKUST-1 catalysts (a) Nanoparticles size (~200 nm) synthesized at room temperature. (b) Micro-particles size (~4 µm) synthesized at 100°C.

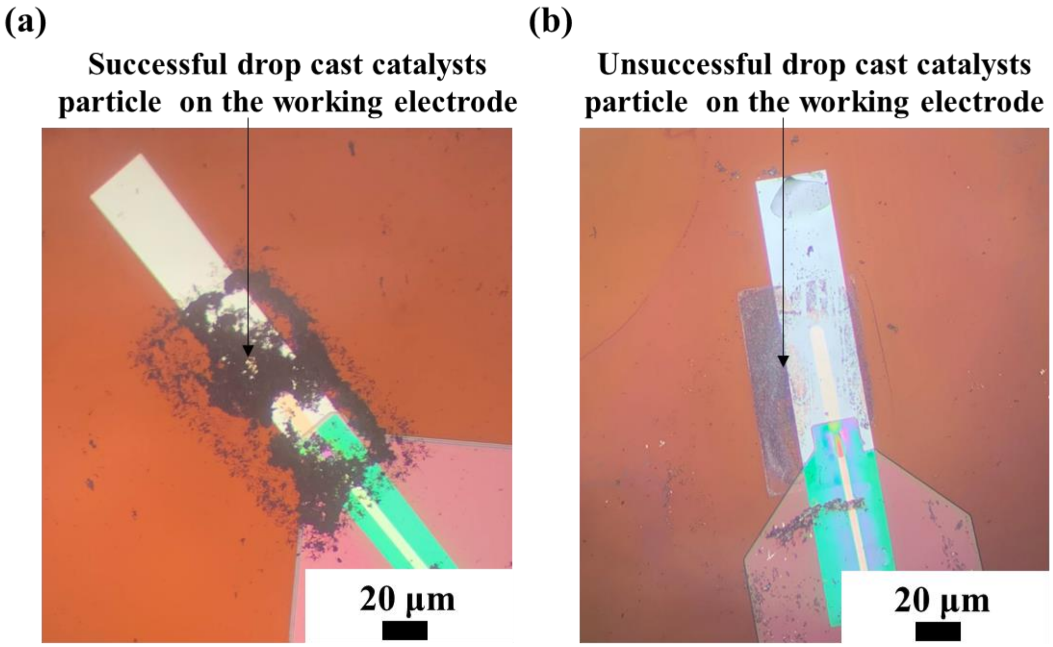

Figure S13. Light microscope images of the drop-cast HKUST-1 on the large E-chip glassy carbon working electrode using the shadow mask: (a) Successful drop-casting of HKUST-1 catalyst achieved by optimizing the catalyst ink with a particle size ~ 200 nm (catalyst synthesized at room temperature). (b) Unsuccessful drop-casting of HKUST-1 occurred when the catalyst ink was distributed around the working electrode.

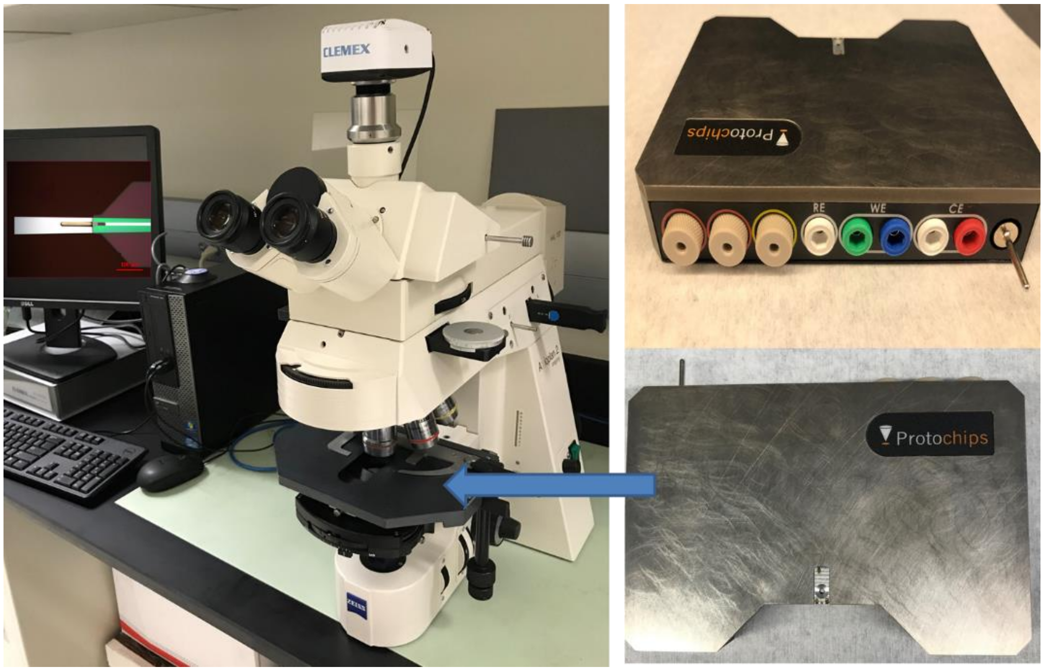

Figure S14. Ex-situ Protochip setup used to visualize bubble formation. The image displays a light microscope setup, consisting of a high-resolution Clemex microscope linked to a computer system, which is used for image analysis and monitoring. The setup includes a Protochips box (top right), equipped with various connection ports labeled for different electrodes (RE, WE, CE), facilitating precise electrochemical measurements.

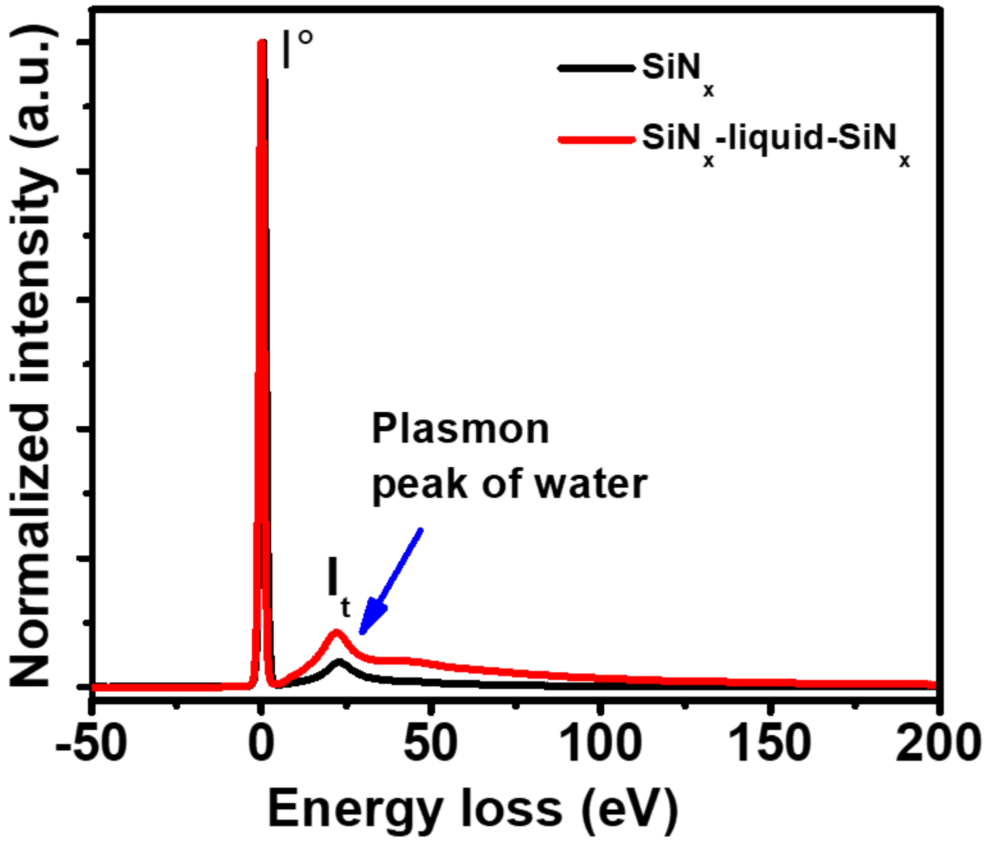

Figure S15. STEM-EELS measurements of the zero-loss peak for a dry 50 nm SiN_x window compared to thin film liquid-filled LP-TEM electrochemical cell (SiN_x-liquid-SiN_x) at an applied potential of -1.0 V vs. Pt in CO_2 saturated 0.1M $KHCO_3$.

Calculation of liquid thickness for in situ LP-TEM using EELS:

The relative liquid thickness (t) in terms of the mean free path of the electrons (λ) can be estimated from the plasmon scattering peak in the EELS spectra by using the Beer–Lambert's law equation:

$t/\lambda=-\ln (I°)/I_t)$

Where:

I°: the number of unscattered electrons in the zero-loss peak

I_t: the total number of incident electrons

From this equation, the relative thickness of the liquid (t liquid/λ liquid) can be estimated by subtracting the relative thickness of the two dry 50 nm SiN_x membranes (t/λ) SiN_x) from the total relative thickness of the entire liquid-filled electrochemical cell ((t/λ)SiN_x-liquid-SiN_x) as shown in the following equation:

$t_{liquid}/\lambda_{liquid}$ =(t/λ)_(SiN_x-liquid-SiN_x)- 〖2(t/λ)〗 _(SiN_x)

(t/λ)SiN_x was calculated to be 0.38 and (t/λ)SiN_x-liquid-SiN_x was calculated to be 1.23, leading to a calculated **$t_{liquid}/\lambda_{liquid}$** value of **0.47**. Assuming the inelastic mean free path of the water to be 106 nm[3,] the liquid thickness within the LP-TEM electrochemical cell was calculated to be ca. 50nm.

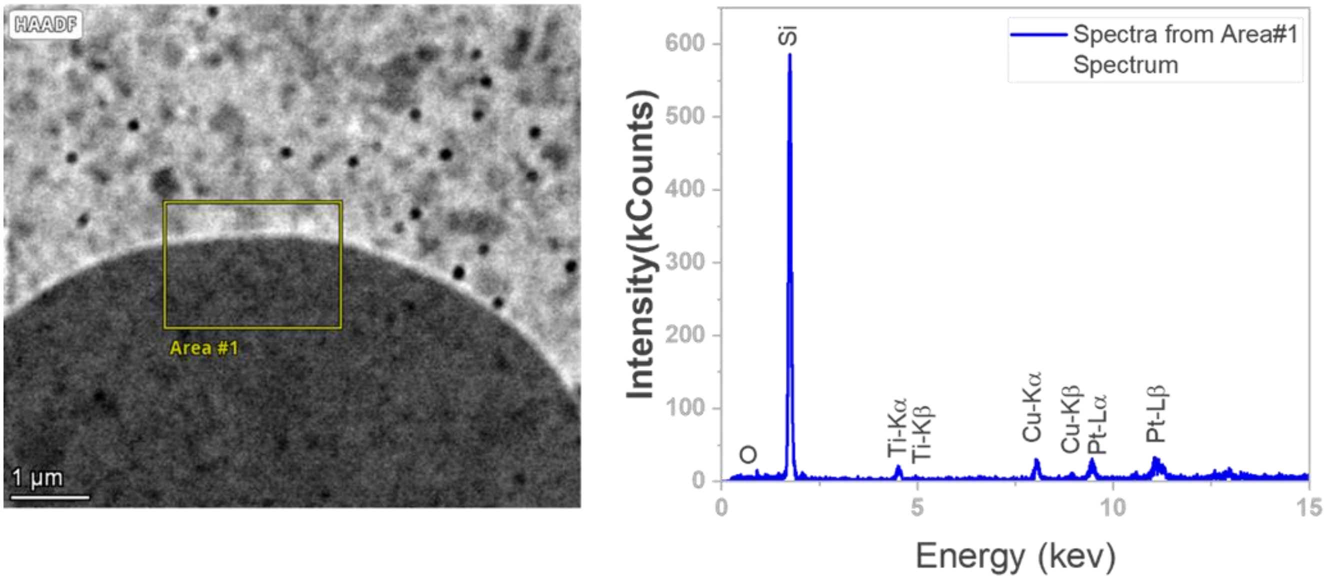

Figure S16. HAADF-STEM and EDX-spectrum from Area 1 of the Pt connector/Cu thin film interface in dry cell. Note that the source of Ti peak in EDX spectrum comes from the lid of the in situ TEM holder.

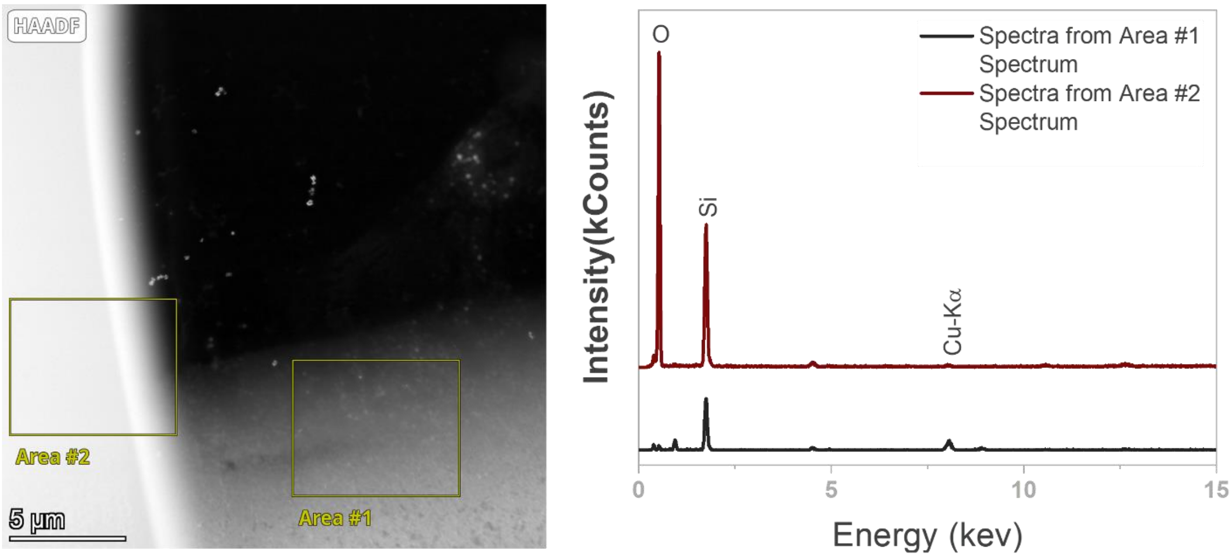

Figure S17. HAADF-STEM and EDX spectrum from Area 1 and 2 indicated in the HAADF-STEM image of the Cu thin film/electrolyte/glassy carbon electrode interface.

CrysTBox ringGUI - Analysis Report

Partial results of individual analysis steps follow.

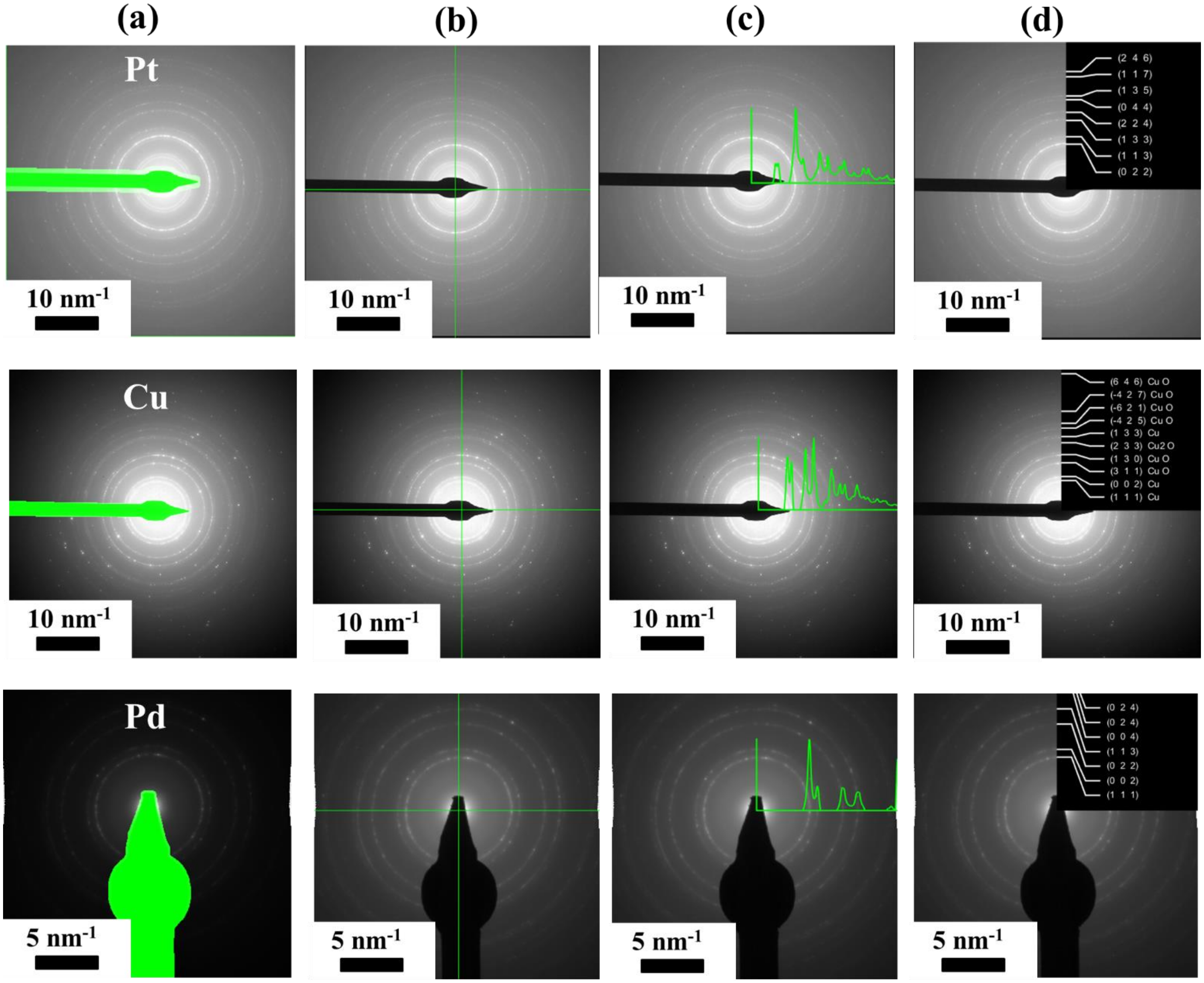

Figure S18. Results of individual steps performed for analyzing the data extracted from CrysTBox ring GUI software: (a) Beam stopper detection. (b) Rings center. (c) Diffractogram profile. (d) Ring identification. Individual lattice vectors are crystallographically identified and the zone axis was calculated.

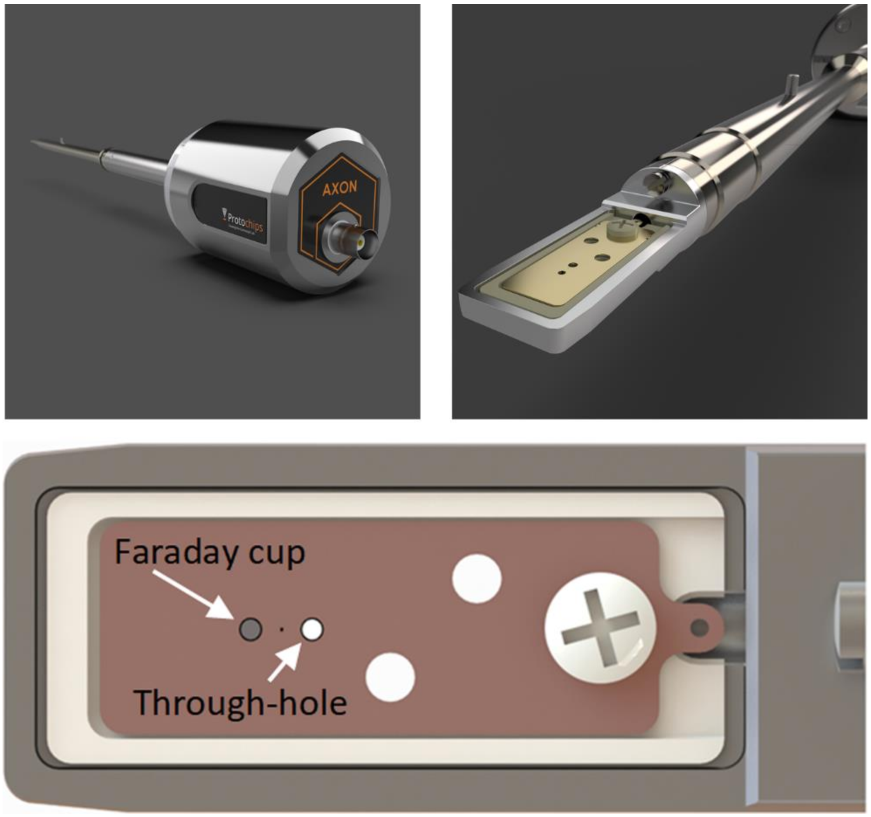

Figure S19. AXON Dose TEM holder. The tip includes two areas—a Faraday cup for collecting beam current and a through-hole that allows the beam to pass through the tip so that its area can be measured using the camera. The Dose holder is connected to a pico-ammeter to accurately measure small currents. A software-driven automated workflow correlates beam current and beam area across various microscope settings.

C) Supplementary Tables (S1 to S3)

Table S1. Crystallographic ring identification for Pt connector.

Ring identification				
Plane	Radius [1/nm]		d-spacing [nm]	
	theor.	measured	theor.	measured
(0 2 2)	7.215	7.247	0.139	0.138
(1 1 3)	8.461	8.455	0.118	0.118
(1 3 3)	11.120	11.113	0.090	0.090
(2 2 4)	12.497	12.441	0.080	0.080
(0 4 4)	14.431	14.495	0.069	0.069
(1 3 5)	15.092	15.099	0.066	0.066
(1 1 7)	18.218	18.239	0.055	0.055
(2 4 6)	19.090	19.085	0.052	0.052

Table S2. Crystallographic ring identification for sputtered Cu thin film.

Ring identification				
Plane	Radius [1/nm]		d-spacing [nm]	
	theor.	measured	theor.	measured
(1 1 1) Cu	4.791	4.816	0.209	0.208
(0 0 2) Cu	5.533	5.465	0.181	0.183
(3 1 1) Cu O	7.716	7.734	0.130	0.129
(1 3 0) Cu O	9.063	9.077	0.110	0.110
(2 3 3) Cu2 O	11.010	11.022	0.091	0.091
(1 3 3) Cu	12.058	12.041	0.083	0.083
(-4 2 5) Cu O	13.430	13.430	0.074	0.074
(-6 2 1) Cu O	14.167	14.218	0.071	0.070
(-4 2 7) Cu O	16.233	16.209	0.062	0.062
(6 4 6) Cu O	22.397	22.368	0.045	0.045

Table S3. Crystallographic ring identification for deposited Pd.

Ring identification				
Plane	Radius [1/nm]		d-spacing [nm]	
	theor.	measured	theor.	measured
(1 1 1)	4.453	4.467	0.225	0.224
(0 0 2)	5.141	5.125	0.194	0.195
(0 2 2)	7.271	7.265	0.138	0.138
(1 1 3)	8.526	8.558	0.117	0.117
(0 0 4)	10.283	10.391	0.097	0.096
(0 2 4)	11.496	11.355	0.087	0.088
(0 2 4)	11.496	11.849	0.087	0.084

References

1. Abdellah, A. M.; Ismail, F.; Siig, O. W.; Yang, J.; Andrei, C. M.; DiCecco, L.-A.; Rakhsha, A.; Salem, K. E.; Grandfield, K.; Bassim, N., *Nature Communications* **2024,** *15*, 938.
2. Li, B.-C.; Lin, J.-Y.; Lee, J.; Kwon, E.; Thanh, B. X.; Duan, X.; Chen, H. H.; Yang, H.; Lin, K.-Y. A., *Colloids and Surfaces A: Physicochemical and Engineering Aspects* **2021,** *631*, 127639.
3. Lin, K.-Y. A.; Yang, H.; Petit, C.; Hsu, F.-K., *Chemical engineering journal* **2014,** *249*, 293-301.
